# Integrated Behavioral Health: A Curriculum for Residents in Rural and Community Psychiatry

**DOI:** 10.15766/mep_2374-8265.11468

**Published:** 2024-12-20

**Authors:** Poonè Haghani Tehrani, Kelsey J. Sala-Hamrick, Sushilla Knottenbelt, John P. Sánchez, Julie G. Salvador

**Affiliations:** 1 Associate Professor, Department of Psychiatry and Behavioral Sciences, University of New Mexico Health Sciences Center; 2 Clinical Psychologist, Department of Psychiatry and Behavioral Sciences, University of New Mexico Health Sciences Center; 3 Senior Lecturer III, Department of Chemistry and Chemical Biology, University of New Mexico; 4 Dean, Universidad Central Del Caribe School of Medicine

**Keywords:** Integrated Psychiatry, Primary Care Integration, Teaching Curriculum, Resident Training, Rural Behavioral Health, Underserved Populations, Case-Based Learning, Community-Based Health Care, Rural Health Care & Training, Integrated Behavioral Health

## Abstract

**Introduction:**

Mental health and substance use disorders are common in the United States; however, only a portion of adults with these conditions receive treatment. Recent recommendations include using integrated behavioral health (IBH) models to increase patient access to care. Despite IBH's effectiveness, few psychiatry residents are trained in it. Considering the scarcity of evaluated curricula on IBH, we created a curriculum to teach different IBH models to psychiatry residents.

**Methods:**

The curriculum was developed using the constructivism theoretical framework and aligned with the principles of competency-based medical education. The learning activities allowed learners to apply knowledge relevant to IBH models to critically appraise a clinical scenario while practicing different components of IBH, such as electronic consults. More specifically, the curriculum assignment prompted residents to examine a clinical practice, identify the model, make recommendations for changes, and discuss the advantages and barriers of the proposed changes. We employed Kirkpatrick model levels 1 and 2b to evaluate the curriculum.

**Results:**

Thirty-three residents participated in this curriculum. Eleven residents completed the assignment, which was qualitatively coded to evaluate their learning. Results indicated that the participants were able to compare different IBH models and critically appraise clinical practice using knowledge of those models. Twenty-two additional residents completed an anonymous retrospective pre- and postrotation survey on their perceived level of proficiency. Survey results showed improved perceived level of proficiency at rotation completion.

**Discussion:**

The developed curriculum was successful in teaching residents to acquire and apply knowledge relevant to IBH.

## Educational Objectives

By the end of this activity, learners will be able to:

1. Compare different models of behavioral health integration.
2. Critically appraise clinical practice, using their knowledge of different models of behavioral health integration.

## Introduction

Mental health and substance use disorders are prevalent in the United States. According to the 2021 National Survey on Drug Use and Mental Health, one in three US adults experience a substance use disorder or mental illness each year.^1^ Despite recent national attention, significant unmet needs for mental health care continue to exist. In 2016, only 1.5% of adults with a substance use disorder received any treatment, and less than 1% received treatment in a specialty facility.^2^ Among adults with mental illness, only 14.4% received any type of mental health treatment.^2^ Numerous barriers contribute to this gap in services, including lack of available providers, treatment cost, not knowing where to find treatment, lack of perceived need, and stigma.^2,3^ Given this remarkable void in fulfilling needs, numerous policy and practice organizations have recommended primary care clinics as key targets for identifying and providing care for these patients.^4–7^ Primary care clinics see 83.4% of US adults each year and can serve as a key entry point to services, where providers can identify needs, provide care, and refer to further behavioral health (BH) treatment and support services.^3^

Strong evidence exists for the use of integrated behavioral health (IBH) models in primary care clinics in order to address current gaps in mental health and substance use treatment services, improve patient physical and mental health outcomes, and reduce health care costs. Integrated care refers to “any attempt to fully or partially blend behavioral health services with general and/or specialty medical services.”^8^ Key components of IBH models include a multidisciplinary care team (including psychiatrists, psychologists, case managers, nurses, primary care providers [PCPs], and patients and families), a clinical setting where collaboration among team members is possible, and implemented clinical processes (screenings, risk protocols, identification of level of care needed, treatment, monitoring, and referral processes).^9^

A large body of research demonstrates the effectiveness of BH care integration. IBH models have been shown to increase access to BH care, improve quality of care, improve patient and provider satisfaction, reduce stigma, and decrease cost of care.^10,11^ These models can leverage an established and trusting relationship with a PCP to promote the early identification of mental health and substance use needs and reduce patients’ fear of stigma associated with seeking BH care. To this end, many studies have shown that integrated care is associated with lower no-show rates.^10–12^ Moreover, utilizing IBH models promotes the use of biopsychosocial and patient-centered approaches to care, which are particularly important when treating patients from historically underserved and socioeconomically disadvantaged backgrounds. For example, utilizing multidisciplinary treatment teams allows for holistic assessment and treatment of patients, including the cultural, social, psychological, and biological aspects of their lives that may be impacting their current health, functioning, and ability to adhere to treatment.^10,12^

Psychiatrists can play a large role in implementing IBH. According to the Institute of Medicine, psychiatrists and psychiatric organizations are tasked with improving the quality of mental health and substance use care in their daily practice.^13^ In alignment with these recommendations, key roles of psychiatrists on IBH teams include ensuring care is patient centered, assessing for biopsychosocial and cultural factors that may impact patients’ conditions and treatment, implementing the use of valid and reliable questionnaires to assess and track care outcomes, and ensuring effective collaboration with PCPs (e.g., consulting with PCPs on diagnoses, how mental health and substance use conditions may impact other comorbidities, and recommending treatment plans).^13^ In addition to IBH's positive impact on patient care, both primary and BH providers report a high level of satisfaction with and value for IBH services.^10–12^ One study showed that IBH provides a context for warm handoffs where BH providers triage patients and make recommendations, while PCPs reported that it frees up their time.^12^

Despite the effectiveness of integrated primary care models, their implementation has been slow. For example, rates of PCPs utilizing evidence-based IBH models are low, less than half of PCPs colocate with a BH provider, and mental health screening rates in primary care clinics are less than 5%.^3,14–16^ One potential reason for this lack of uptake is the relatively limited training that psychiatry residents and other clinicians receive on IBH models and their advantages. While some training programs exist in pediatric primary care settings,^17,18^ to date there are no peer-reviewed and published curricula to support residents in learning and implementing IBH models.

Currently, there is a small number of studies on integrated care education for various groups of learners, some of which have shown positive evaluation from learners. These include a brief collaborative care curriculum for first- to fourth-year psychiatry residents,^19^ a brief rotation for third-year psychiatry and family medicine residents in the family medicine clinic at five different institutions (no data on the outcome of this curriculum were provided),^20^ a competency-based curriculum on IBH training for first- to third-year family medicine residents across multiple programs,^21^ and a comprehensive program designed for master's-level social work students.^22^ These studies offer promising data on curricula related to IBH; however, they vary with respect to the targeted group of learners, curriculum design, and evaluation. Additionally, some of them have not assessed the outcomes of their curricula.^23^ Therefore, it is not possible to draw definitive conclusion regarding IBH curriculum design. Notably, the existing publications primarily concentrate on developing clinical skills as consultants in integrated settings, without placing significant emphasis on familiarity with various IBH models. Moreover, most of these studies have not published their curricula, including details of implementation.

To address these gaps, we created a curriculum in the context of newly developed IBH services at a university-affiliated hospital to teach different models of IBH to psychiatry residents. The goal was for residents to acquire and apply knowledge relevant to IBH.

## Methods

### Curriculum Development

The curriculum was developed using the constructivism theoretical framework. This view of learning assumes that learners, as active participants in their learning, constantly strive to make meaning of their learning experiences. This process is affected by the individual learner's existing knowledge foundations and cognitive operations, as well as the learning activities in which they engage.^24^ This approach aligns with the Accreditation Council for Graduate Medical Education (ACGME) competency-based and learner-centered guidelines.^25^

The curriculum-targeted learners were second-year psychiatry residents, who participated in the learning activities 1 day a week as part of a preexisting 1-month rural and community psychiatry rotation. The learners had no prior formal training in IBH in their residency training.

To familiarize themselves with foundational knowledge about IBH, facilitators used the background for facilitators (Appendix A) and the learner guide (Appendix B).

The curriculum's learning activities were organized in four main categories: (1) acquiring information and ideas, (2) experience, (3) reflection, and (4) assessment of the learners and the curriculum (Figure). A literature review of different IBH models and a clinical scenario provided the educational context for acquiring and applying knowledge relevant to IBH. While not required, we offered an optional 4-day rotation as an opportunity to observe and experience practice of IBH within a family practice clinic.^26^

**Figure.**
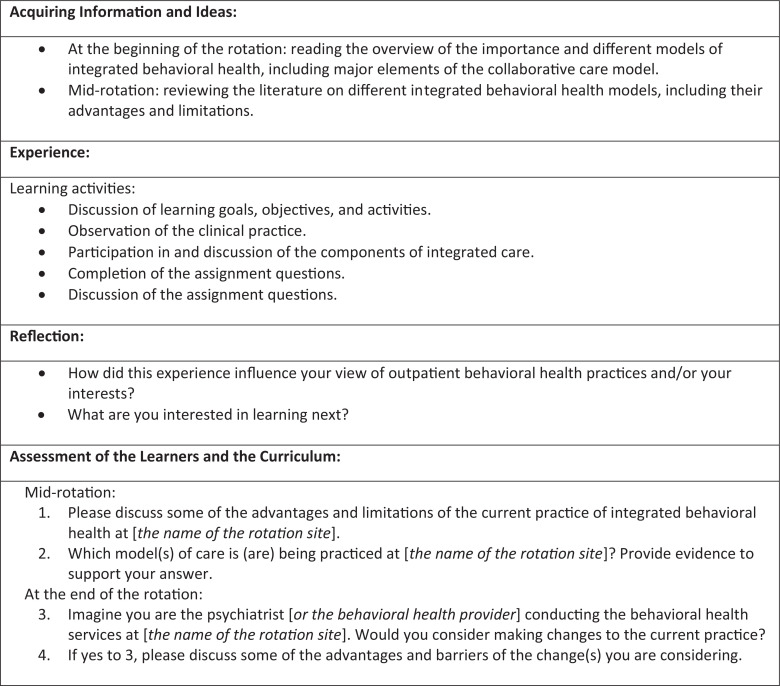
Curriculum outline.

As outlined in the Figure, for acquiring information and ideas, the residents reviewed the learner guide (Appendix B). This document included the curriculum's learning goals and objectives; an overview of the importance and different models of IBH, including major elements of the collaborative care model; and the curriculum assignment and citations for the content on IBH. As described in the session 1 facilitator guide (Appendix C), the facilitator provided this document to the participating resident right before or on the first day of the rotation. The second phase of acquiring information and ideas took place mid-rotation, when the resident reviewed the literature on different IBH models, including their advantages and limitations. This is further explained in conjunction with the learning activities described in the following paragraphs.

For experience, we defined several learning activities to be completed in sessions 1-4, along with a reflection exercise in session 4. Session 1 (Appendix C) began with a discussion of the learning goal and objectives, providing an opportunity for residents to participate in planning their learning. This was followed by an overview of the learning activities. Additionally, the communication of the rotation's expectations, ground rules for mutual feedback (e.g., timing, frequency, and aims for feedback), introduction to the clinic staff and providers, and orientation to the clinic physical space took place at the beginning of this session.

In addition to partaking in setting goals, the resident observed the clinical practice and participated in components of integrated care during session 1. These components included electronic consults (E-consults), curbside consults, and warm handoffs, which the facilitator discussed with the resident as described in Appendix C. Appendix C also provided examples for simulated E-consults to be used if the integrated team did not receive an E-consult during the resident's time at the rotation site.

In session 2, the main learning activity was to answer two questions referred to as assignment questions 1 and 2 in the curriculum outline and the supporting material. These questions prompted the resident to discuss the advantages and limitations of the observed integrated practice and to identify the model(s) of this practice. The session 2 facilitator guide (Appendix D) included instructions for leading this session.

In session 3, the primary focus was to review the resident's responses to assignment questions 1 and 2; discuss the details, including different models of IBH; and address possible misconceptions. The session 3 facilitator guide (Appendix E) provided details for conducting this discussion.

Session 4 contained three key activities: answering assignment questions 3 and 4, reviewing and discussing the resident's response to these questions, and the reflection exercise. Assignment questions 3 and 4 prompted the resident to examine whether they would consider making changes to the practice they had observed and, if so, to discuss the advantages and barriers of the changes they were considering. The detailed guide for conducting this discussion was included in the session 4 facilitator guide (Appendix F).

Appendix G provided supplementary PowerPoint slides for Appendices C–F to enhance teaching.

### Curriculum Implementation

The curriculum was implemented at a family practice clinic (University of New Mexico Sandoval Regional Medical Center) where psychiatric services were integrated to improve the quality of and access to care for the underprivileged patient population of the clinic within the limits of the existing resources. The curriculum was executed in 2017 with ongoing iterations (except in 2020, due to the COVID-19 pandemic) guided by the on-site psychiatrist. It could be implemented in any IBH setting, regardless of the model of integration. When implementing this curriculum in an IBH setting, it was crucial not to disclose the specific IBH model. This approach allowed learners the opportunity to identify the model as part the curriculum assignment. Additionally, Appendix H offered a supplementary simulation scenario for implementation of this curriculum outside of a clinical setting. Furthermore, the curriculum could be completed in two instead of four sessions. The instructions for this were included in Appendix C.

### Curriculum Evaluation

To evaluate the curriculum's effectiveness in accomplishing the Educational Objectives (EOs), we employed levels 1 and 2b of the Kirkpatrick model, a four-level model originally created for industry training evaluation but widely used in educational assessments. These include medical education, which has adopted a modified version of the model. Consistent with Kirkpatrick level 1, we gauged learners’ perception of the curriculum's effectiveness. Based on Kirkpatrick level 2b, we assessed the curriculum's impact on learners’ knowledge and skills.^27^ Data from the first seven classes of residents who participated in the curriculum were used to evaluate the curriculum. An average of 9.6 residents per year participated in this rotation. Only residents who participated in at least two sessions of the rotation and completed the assignment were included (total of 33 residents).

For the first three classes of residents who participated in the rotation (*n* = 11), residents’ responses to the curriculum assignment were used to assess the effectiveness of the curriculum. This was consistent with the principle of assessment for learning instead of assessment of learning, which fosters a growth mindset.^28^ An inductive approach to content analysis was used to code data focused on the EOs. NVIVO qualitative software was used to code resident responses and to help identify overarching themes that emerged directly from resident responses to the curriculum assignment.^29,30^ Using the EOs and resident assignment as a coding guide, the senior author, Julie G. Salvador, a trained qualitative researcher, conducted initial coding of the written responses and developed preliminary theme labels. Julie G. Salvador and Poonè Haghani Tehrani (lead author) then jointly reviewed the coded statements and theme labels to come to a final agreement on placement of codes and meaningful theme labels. Themes were organized under each area according to the focus of learner responses relating to advantages, limitations, and suggestions for improvement identified by learners at the provider, patient, or general health care system level.

For the last four classes of residents who participated in the rotation (*n* = 22), an anonymous retrospective pre- and postrotation survey (Appendix I) was implemented. The survey assessed the residents’ perceived level of proficiency in each of the curriculum EOs before and after the rotation. University of New Mexico Institutional Review Board (IRB) exemptions were obtained.

## Results

Thirty-three residents participated in this rotation. To help evaluate the curriculum, 11 residents completed the qualitative curriculum assignment, and the remaining 22 completed a quantitative survey. Results from both the qualitative and quantitative evaluation processes are presented below.

### Qualitative Evaluation

The resident responses to the assignment were qualitatively analyzed to evaluate the accomplishment of the curriculum EOs: (1) how well residents were able to compare different models of BH integration and (2) how well residents were able to critically appraise clinical practice, using their knowledge of different models on BH integration. Regarding EO 1, the results of this qualitative analysis showed that residents were able to compare different models of IBH. As part of the assignment, residents were prompted to review the literature on different models of IBH and discuss what model or models were being practiced at the rotation site. In response, residents identified general features of IBH models. Collaborative care was referred to most often, with learners describing this model as led by a PCP and supported by care managers as liaisons between the patient, the PCP, and the BH professional. Fundamental aspects of this model identified included that it is evidence based and team driven, uses multidisciplinary teams and problem-based referral, is population based, and uses measurement-guided treatment. A second model identified was colocation. Learners described this model as patients seeing both the PCP and a BH expert, with frequent communication between providers on shared goals of the patient. Regarding EO 2, residents demonstrated the ability to critically appraise clinical practice using their knowledge of different models of BH integration. In their responses to the curriculum assignment, residents discussed numerous advantages, limitations, and recommendations for improvement of the integrated model at the rotation site. These responses are narratively described below and reflected in Tables 1–3. For each area (advantages, limitations, and recommendations for improvement), responses focused on three general categories: impact on clinicians and providers, impact on patients, and impact on health care system and processes.

**Table 1.**
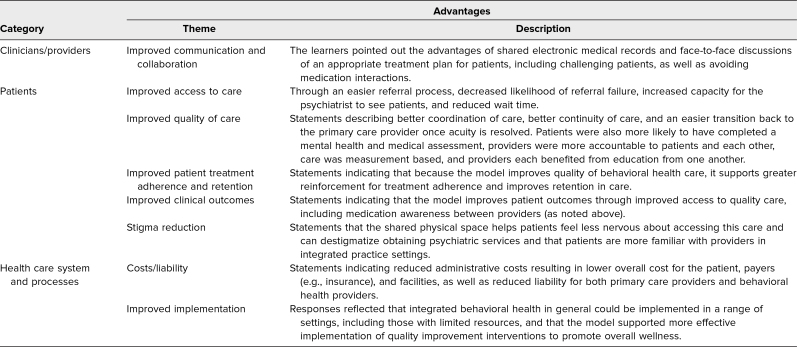
Residents’ Responses on Advantages of the Integrated Practice at the Rotation Site (*N* = 11)

Table 1 shows residents’ responses regarding the advantages of integrated practice. At the clinician/provider level, these advantages included improved communication and collaboration resulting from integrated care, such as the ability to have curbside consultations and better support between the PCP and psychiatrist. At the patient level, themes included enhanced access to and quality of care, and improved patient outcomes. Residents also reported that integrated care helped reduce stigma for patients about receiving psychiatric treatment. For the health care system, residents reported that integrated care helped reduce costs and liability for providers and that the model could be implemented in a wide range of venues.

Table 2 presents residents’ responses regarding the limitations of integrated practice. At the clinical/provider level, these limitations included challenges with time management and spending time with lower-acuity patients who could be effectively managed by midlevel BH providers. At the patient level, residents reported that IBH programs worked best in contexts where the patient had multiple comorbidities and psychiatric issues were not the primary diagnosis. At the health care system level, residents noted the need to develop protocols to support implementation.

**Table 2.**
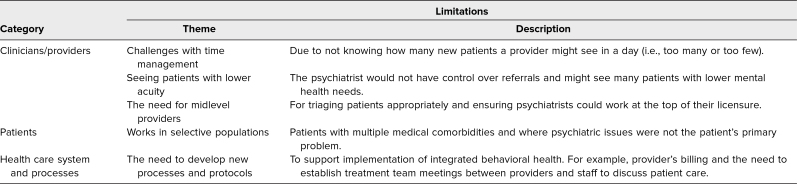
Residents’ Responses on Limitations of the Integrated Practice at the Rotation Site (*N* = 11)

Table 3 presents residents’ recommendations for improvement of integrated practice. At both the clinical/provider level and the patient level, more education was recommended, aimed at helping PCPs to identify and refer patients appropriately and to enhance awareness and comfort for patients to access BH services. At the health care system level, recommendations included adding other BH services (e.g., group therapy) and financial incentives to encourage the implementation of IBH.

**Table 3.**
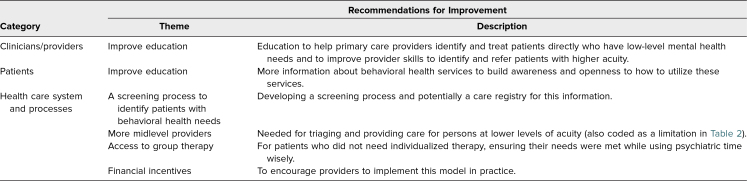
Residents’ Responses on Recommendations for Improvement of the Integrated Practice at the Rotation Site (*N* = 11)

### Quantitative Evaluation

Twenty-two residents completed a retrospective pre- and postrotation anonymous survey (survey response rate: 100%) where they rated their proficiency in comparing different models of IBH (EO 1) and in critically appraising clinical practice using their knowledge of different models of IBH (EO 2) on a 5-point scale (1 = *not at all proficient*, 5 = *extremely proficient*). The mean rating of resident proficiency in comparing different models of IBH increased from 1.9 to 4.1. The mean rating of their ability to critically appraise clinical practice using their knowledge of different models of IBH improved from 1.6 to 3.9.

## Discussion

Considering the evidence for the effectiveness of IBH in fulfilling health care goals of improving quality of care and health of populations while reducing the cost of care,^8–12,31^ it seems reasonable to anticipate an increasing demand for psychiatrists, other BH professionals, family medicine physicians, and pediatricians to be part of developing or operating in IBH settings. Therefore, it is crucial that these groups of health care professionals are familiar with IBH models. This is reflected in the inclusion of “understanding of models of integrated multidisciplinary mental health and primary care” and serving “as a leader of integrated teams of implementation projects” in the ACGME milestones for psychiatry residents.^25^

To address the gap in learners’ familiarity with different IBH models, we developed and evaluated the curriculum detailed herein. The results revealed the effectiveness of our brief and innovative educational resource. Given the constraints of the overarching rotation, including considerations of timing and duration, the curriculum's learning activities were designed to cater to early-stage learners while serving as an assessment for learning. Significantly, this design was shown to be effective in achieving the curriculum EOs, as the learners were able to acquire adequate knowledge of different models of IBH, apply that knowledge to a comparison of various existing models, and critically appraise the rotation site model. Our curriculum design, tailored to early-stage learners, provides them with an opportunity to explore their interest in IBH. Additionally, the curriculum's brevity enhances its feasibility.^21^

We encountered barriers during the curriculum's implementation and data collection. One challenge was completing IRB approval after the enrolled residents had graduated. Consequently, we were unable to obtain consent to include quotes from their responses to the curriculum assignment. Another obstacle arose from the fact that second-year psychiatry residents’ training in our program predominantly focuses on inpatient and psychiatric emergency services. As a result, some residents found it challenging to shift from fast-paced, high-acuity settings to outpatient care, and some reported discomfort commuting to the rotation site. Moreover, some classes were encouraged to take leave during their rural and community rotation, which encompassed our curriculum. Also, with the onset of the COVID-19 pandemic, it became more likely that backup on-call residents would be called in. These factors impacted the number of residents who completed this rotation and, therefore, our sample size. A unique challenge in teaching different IBH models is the use of the term *integrated* to refer to higher levels of integration, rather than encompassing the full spectrum of integrated care models. This deserves attention in future studies on IBH models and the associated educational experiments.

One essential element of this curriculum's implementation was its location at a newly developed university-affiliated hospital in an underserved community. While this provided institutional support for the development of integrated services in the family practice clinic, including support for faculty time, dedicated clinical space and staff, integration of the faculty psychiatrist and residents into the clinic's physical space and providers’ workstations, and acceptance of the residents in their role, it also presented challenges. Many academic institutions partner with nonacademic facilities, offering learning opportunities but potentially causing implementation challenges due to the separation of leadership and stakeholders. For instance, due to resource and workforce limitations, it was challenging to garner support for residents’ quality improvement projects related to this curriculum. Also, because of the distribution of funding under the Office for Graduate Medical Education, interested residents could not return to this rotation site for an elective rotation to expand their clinical experience in integrated care.

This project had some limitations. Firstly, responses to the curriculum assignment were obtained either in person verbally or via email, potentially impacting how residents responded. Based on their preference and learning style, learners were given the choice of an in-person verbal discussion of their responses to the assignment or an email exchange. For those who discussed their responses verbally, accuracy was ensured by repeating their responses back to them and documenting them instantly. Another limitation was the variation in the number of days residents participated in the rotation, ranging from 2 to 4 days. Despite this, all residents completed the assignment and were able to critique the practiced model at the rotation site. Additionally, we had a small sample size (*n* = 33). While having a larger group of participants would have been beneficial, the rotation was part of a 1-month rural and community psychiatry track, which was an elective during the first few years of this project. Therefore, only one resident per month could opt in if they chose to participate in the rural and community track.

Our goal is to make this curriculum available to educators as part of global efforts toward IBH training. Given the scarcity of published, peer-reviewed curricula, this publication contributes to the development, implementation, and standardization of integrated care curricula. This curriculum is adaptable for utilization by psychiatry, family medicine, and pediatrics residency programs, as well as programs in BH training, including clinical psychology, social work, BH counseling, nurse practitioner, and physician assistant training programs. It can be implemented in primary care and specialty care clinics, in academic and nonacademic institutions, spanning urban and underserved areas, regardless of the type of integrated model practiced, as well as outside of clinical settings.

## Appendices


Background for Facilitators.docxLearner Guide.docxSession 1 Facilitator Guide.docxSession 2 Facilitator Guide.docxSession 3 Facilitator Guide.docxSession 4 Facilitator Guide.docxFacilitator Guide Slides.pptxSimulation Scenario.docxEvaluation Survey.docx
*All appendices are peer reviewed as integral parts of the Original Publication.*
