# Supplementary material for: Integrated Behavioral Health: A Curriculum for Residents in Rural and Community Psychiatry
Source: MedEdPORTAL. 2024 Dec 20;20:11468. doi: 10.15766/mep_2374-8265.11468 (PMC11659397; doi:10.15766/mep_2374-8265.11468)
Supplement: Supplementary file 1 — Background for Facilitators.docxLearner Guide.docxSession 1 Facilitator Guide.docxSession 2 Facilitator Guide.docxSession 3 Facilitator Guide.docxSession 4 Facilitator Guide.docxFacilitator Guide Slides.pptxSimulation Scenario.docxEvaluation Survey.docx [file mep_2374-8265.11468-s001.zip › A. Background for Facilitators.docx]

**Appendix A**

**Background for Facilitators**

This document includes a summary of the foundational information and a glossary of terminology related to integrated behavioral health. Facilitators can use it to prepare for teaching.

**What is integrated Care?**

Integrated care “is a general term for any attempt to fully or partially blend behavioral health services with general and/or specialty medical services” (1).

**The efforts that led to the idea of integrated care started with the Institute of Medicine “Crossing Quality Chasm”** **2001 report:**

“Quality problems are everywhere, affecting many patients. Between the health care we have and the care we could have lies not just a gap, but a chasm.”

In 2014, the United States was ranked the lowest among industrialized countries concerning efficiency, equity, and outcome of care despite the highest healthcare expenditure which rose to $3.03 trillion in 2014 and to $3.8 trillion in 2019 per Centers for Medicare and Medicaid Services’ report.

Recommendation: Care that was safer, more reliable, more responsive, more integrated, and more available; and addressed the full array of preventive, acute, and chronic services (2–4).

**The Institute for Healthcare Improvement suggested Triple Aim Healthcare (5):**

- The patient experience of care (quality and satisfaction).
- The health of populations.
- Reducing the per capita cost of health care.

**Regarding Mental Health and Substance Use Care (6):**

- Access to and sustainability of care was a specific focus in addition to the general recommendations for healthcare.
- Some of the Institute of Medicine recommendations for psychiatrists and psychiatric organizations:
- Pursue patient-centered care.
- Increase use of valid and reliable questionnaires to assess and track outcomes of treatment.
- Use effective mechanisms to link mental health and substance use providers with primary care providers.

**Advantages of Integrated Behavioral Health** (1,7–10)**:**

- Biopsychosocial approach to care.
- Patient-centered approach to care.
- Team-based model of care.
- Reduced stigma.
- Reduced cost of care.
- Efficient use of resources as it improves access to care, quality of care, and patient and provider satisfaction.
- Reduction in no-show rates and wait times.

**Levels of Integration (11,12):** **Behavioral Health Consultation (13):**

A behavioral health consultant or clinician is:

- A behavioral health professional (e.g. psychologist, psychiatrist).
- A generalist (helps with the care of patients of any age and with any condition).
- A routine member of the primary care team.
- An educator (provides formal and informal education to PCPs and patients).
- Shares clinic space and resources with the PCPs.
- Accessible (via providing warm hand-offs and curbside consults).
- Provides team-based services.
- Uses a biopsychosocial approach to care.
- Provides high-volume services (provides care to a large percentage of the clinic population).
- Uses 15-30min visits focused on specific symptoms and functional improvement.

**Collaborative Care Model- Core Principles (14):**

- Patient-Centered Team Care.
- Population-Based Care.
- Measurement-Based Treatment to Target.
- Evidence-Based Care.
- Accountable Care.

**Collaborative Care Model (14):**

**Glossary**

**Integrated care:** “Is a general term for any attempt to fully or partially blend behavioral health services with general and/or specialty medical services” (1). There are different elements of care that can be the focus of integration. Some examples of those elements are: providers co-location, shared access to medical records, agreed referral criteria, single point entry, professional role change, working pattern change, joint assessment and treatment, multidisciplinary teams and meetings, financial integration, presence of a case-coordinator/manager, patient education(15). Depending on what combination of these elements are included in the integration, different models of care form (e.g. coordinated care, co-located care, integrated care, reverse Integrated care, Collaborative Care Model, BH consultation).

**Integrated behavioral health care:** “Blends care in one setting for medical conditions and related behavioral health factors that affect health and well-being”(16).

**Coordinated care:** When the primary care provider (PCP) refers the patients to another practice site. The communication between the PCP and the behavioral health provider is from zero to periodic communication (17).

**Co-located care:** “Co-location refers to services that are located in the same physical space (e.g. office, building, campus), though not necessarily fully integrated with one another” (18).

**Reverse integrated care:** “The reverse of traditional integrated care settings. In this model, PCPs are co-located in the mental health setting”(19).

**Collaborative Care Model: “**Is a specific type of integrated care that treats common mental health conditions such as depression and anxiety that require systematic follow-up due to their persistent nature. Based on [principles of effective chronic illness care](https://aims.uw.edu/collaborative-care/principles-collaborative-care), Collaborative Care focuses on defined patient populations tracked in a registry, measurement-based practice and treatment to target. Trained primary care providers and embedded behavioral health professionals provide evidence-based medication or psychosocial treatments, supported by regular psychiatric case consultation and treatment adjustment for patients who are not improving as expected” (14).

**Behavioral health consultation:** A behavioral health consultant is a behavioral health professional (e.g. psychologist, psychiatrist) who is a routine member of the primary care team, shares clinic space and resources with PCPs and supports the PCPs as a generalist (helps with the care of patients of any age and with any condition) and as an educator (provides formal and informal education to PCPs and patients.) The behavioral health consultant is accessible, provides team-based services, uses a biopsychosocial approach, provides high-volume services (provides care to a large percentage of the clinic population), and Uses 15-30min visits focused on specific symptoms and functional improvement (13).

**Electronic consults (E-consults):** “Asynchronous communication between healthcare providers that occurs within a shared electronic health record (EHR) or secure Web- based platform” (20).

**Curbside consults:** “an informal process whereby a physician obtains information or advice from another physician to assist in the management of a particular patient. The consultant is generally unfamiliar with the patient and has not reviewed the patient's chart or examined the patient” (21).

**Warm handoff:** “A warm handoff is a handoff that is conducted in person, between two members of the health care team, in front of the patient (and family if present)”(22).

References:

1. American Psychiatric Association [Internet]. [cited 2021 May 27]. Integrated Care. Available from: https://www.psychiatry.org/psychiatrists/practice/professional-interests/integrated-care

2. CMS Office of the Actuary Releases 2019 National Health Expenditures | CMS [Internet]. [cited 2022 Jul 7]. Available from: https://www.cms.gov/newsroom/press-releases/cms-office-actuary-releases-2019-national-health-expenditures

3. Committee on Quality of Health Care in America. Crossing the Quality Chasm: (317382004-001) [Internet]. American Psychological Association; [cited 2021 May 11]. Available from: http://doi.apa.org/get-pe-doi.cfm?doi=10.1037/e317382004-001

4. Manchikanti L, Ii SH, Benyamin RM, Hirsch JA. A Critical Analysis of Obamacare: Affordable Care or Insurance for Many and Coverage for Few? Pain Physician. :28.

5. Berwick DM, Nolan TW, Whittington J. The Triple Aim: Care, Health, And Cost. Health Aff (Millwood). 2008 May;27(3):759–69.

6. M. Kwan B, B. Valeras A, Brown Levey S, E. Nease D, E. Talen M, 1 Department of Family Medicine, University of Colorado School of Medicine, Aurora, CO, United States; An Evidence Roadmap for Implementation of Integrated Behavioral Health under the Affordable Care Act. AIMS Public Health. 2015;2(4):691–717.

7. Dale H, Lee A. Behavioural health consultants in integrated primary care teams: a model for future care. BMC Fam Pract [Internet]. 2016 Dec [cited 2019 Apr 2];17(1). Available from: http://bmcfampract.biomedcentral.com/articles/10.1186/s12875-016-0485-0

8. Sunderji N, Ion A, Ghavam-Rassoul A, Abate A. Evaluating the Implementation of Integrated Mental Health Care: A Systematic Review to Guide the Development of Quality Measures. Psychiatr Serv. 2017 Sep;68(9):891–8.

9. Muse AR, Lamson AL, Didericksen KW, Hodgson JL. A systematic review of evaluation research in integrated behavioral health care: Operational and financial characteristics. Fam Syst Health. 2017;35(2):136–54.

10. American College of Physicians, Smith CD, Balatbat C, National Academy of Medicine, Corbridge S, University of Illinois at Chicago, et al. Implementing Optimal Team-Based Care to Reduce Clinician Burnout. NAM Perspect [Internet]. 2018 Sep 17 [cited 2019 Mar 5];8(9). Available from: https://nam.edu/implementing-optimal-team-based-care-to-reduce-clinician-burnout

11. SAMHSA-HRSA 2013 Framework for Levels of Integrated Healthcare.pdf.

12. Coates D, Coppleson D, Schmied V. Integrated physical and mental healthcare: an overview of models and their evaluation findings. Int J Evid Based Healthc. 2020 Mar;18(1):38–57.

13. Reiter JT, Dobmeyer AC, Hunter CL. The Primary Care Behavioral Health (PCBH) Model: An Overview and Operational Definition. J Clin Psychol Med Settings. 2018 Jun;25(2):109–26.

14. Collaborative Care | University of Washington AIMS Center [Internet]. [cited 2022 Feb 17]. Available from: https://aims.uw.edu/collaborative-care

15. Baxter S, Johnson M, Chambers D, Sutton A, Goyder E, Booth A. The effects of integrated care: a systematic review of UK and international evidence. BMC Health Serv Res. 2018 Dec;18(1):350.

16. What is Integrated Behavioral Health? | The Academy [Internet]. [cited 2022 Feb 17]. Available from: https://integrationacademy.ahrq.gov/about/integrated-behavioral-health

17. Gerrity M. Evolving Models of Behavioral Health Integration: :58.

18. Co-location of Services Model - Rural Services Integration Toolkit [Internet]. [cited 2022 Feb 17]. Available from: https://www.ruralhealthinfo.org/toolkits/services-integration/2/co-location

19. Maragakis A, Siddharthan R, RachBeisel J, Snipes C. Creating a ‘reverse’ integrated primary and mental healthcare clinic for those with serious mental illness. Prim Health Care Res Dev. 2016 Sep;17(05):421–7.

20. Vimalananda VG, Gupte G, Seraj SM, Orlander J, Berlowitz D, Fincke BG, et al. Electronic consultations (e-consults) to improve access to specialty care: A systematic review and narrative synthesis. J Telemed Telecare. 2015 Sep;21(6):323–30.

21. Kuo D. Curbside Consultation Practices and Attitudes Among Primary Care Physicians and Medical Subspecialists. JAMA. 1998 Sep 9;280(10):905.

22. Warm Handoffs: A Guide for Clinicians. :1.
