# Supplementary material for: Integrated Behavioral Health: A Curriculum for Residents in Rural and Community Psychiatry
Source: MedEdPORTAL. 2024 Dec 20;20:11468. doi: 10.15766/mep_2374-8265.11468 (PMC11659397; doi:10.15766/mep_2374-8265.11468)
Supplement: Supplementary file 1 — Background for Facilitators.docxLearner Guide.docxSession 1 Facilitator Guide.docxSession 2 Facilitator Guide.docxSession 3 Facilitator Guide.docxSession 4 Facilitator Guide.docxFacilitator Guide Slides.pptxSimulation Scenario.docxEvaluation Survey.docx [file mep_2374-8265.11468-s001.zip › B. Learner Guide.docx]

**Appendix B**

**Learner Guide**

**Learners, please review this document at the beginning of this learning experience (before the second session).**

**Goals:**

This learning experience aims to provide psychiatry residents and other interested learners with the educational context to:

- Acquire and apply knowledge relevant to integrated behavioral health.

**Learning Objectives:**

By the end of this learning experience, each 2^nd^-year psychiatry resident (learner) will be able to:

1. Compare different models of behavioral health integration.
2. Critically appraise clinical practice, using their knowledge of different models of behavioral health integration.

**Rotation Overview**

| **Time** | **Activities** |
| --- | --- |
| Session 1 | Discussion of learning goals, objectives, and activities.  Observation of the clinical practice.  Participation in and discussion of the components of integrated care. |
| Session 2 | Completion of the assignment’s questions 1 and 2.  Observation of the clinical practice.  Participation in and discussion of the components of integrated care. |
| Session 3 | Discussion of the assignment’s questions 1 and 2.  Observation of the clinical practice.  Participation in and discussion of the components of integrated care. |
| Session 4 | Completion of the assignment’s questions 3 and 4.  Discussion of the assignment’s questions 3 and 4.  Observation of the clinical practice.  Participation in and discussion of the components of integrated care.  Reflection on the entire learning experience. |

**Integration of behavioral health services: a brief overview of the importance and different models**

Although one clear definition does not exist, integrated care is often used as “a general term for any attempt to fully or partially blend behavioral health (BH) services with general and/or specialty medical services with the goal of meeting the complex needs of patients through person-centered care.^1–3^” Over the last several decades a growing body of research and policy supports the need for integrating healthcare practices due to its ability to improve patient health outcomes and experience, increase access to and quality of care, and reduce cost, time and overall burden of health, particularly for patients with chronic conditions, those from underserved backgrounds, and those with special healthcare needs. ^4–11^ Recent data shows that 52.9 million adults (21.0% of U.S. adults) experience a diagnosed mental illness and 14.2 million adults (5.6% of U.S. adults) have a serious mental health condition.^12–15^ Approximately 17.0 million people (6.7%) in the U.S. have co-occurring substance use and mental health conditions.^12,14^ Despite the high rates of behavioral health conditions, less than half (46.2%) of adults with a mental health condition received mental health services in 2020 and although two-thirds of adults with a serious mental health condition received services, 49.7% reported an unmet need for mental health services within the past year.^12^

Integrated behavioral health professionals can serve a wide variety of roles in healthcare systems, depending on patient population, clinical setting (e.g. outpatient, inpatient, primary care, specialty clinics), and organization structure and integration. Levels of care integration can vary depending on model, but all integration consists of the same ten elements including screening, patient education/self-management, medication, psychotherapy, coordinated care, clinical monitoring, assessment of medication adherence, standardized follow-up, formal stepped care, and supervision.^5(pp.67),16,17^

As integrated care becomes the standard model for high quality treatment, psychiatrists can play an important role in collaborative care teams and as psychiatric consultants. In such roles, psychiatrists can often support primary care providers and other treatment team members in the treatment of psychiatric conditions, serving as consultants and thus have the ability to impact and improve the care of higher numbers of patients.^18^

Depending on how separated the systems providing primary and behavioral health care are and also the amount of communication in between the two systems, there are different types of integration: coordinated care, co-located care, and integrated care. ^1,6,19^

- Coordinated care:
- Separate locations.
- Screening by PCP.
- Referral linkage.
- +/- Routine communication.
- The patient is connected to other resources in the community.

Increase in integration

- Co-located care:
- Same location.
- Separate visits.
- Screening by PCP.
- Referral process.
- Regular communication.
- Decreases no show rate.
- Integrated care:
- A true team.
- Frequent communication.
- +/- a database to track the progress of these patients.
- Easiest patient access.
- Potential challenges: Documentation, coding, billing, reimbursement.

Collaborative Care (CoMC) Model:

This model was originally developed by Katon and colleagues at the University of Washington.^20–22^ Among different models of delivering integrated care, Collaborative Care Model in particular has the most evidence supporting its effectiveness and efficiency in delivering integrated care. Research has consistently shown that this model reduces cost, inequities, and stigma related to BH, improves access to care and clinical outcome, and increases patient and provider satisfaction in various community settings (e.g. urban, rural, among veterans).^23^ The core elements of CoCM model include being Team-Driven, Population-Based, Measurement-Guided, and Evidence-Based. These four elements combined together lead to another guiding principal of CoCM model: Accountability and Quality Improvement.^23^

Also, Collaborative Care Model is patient-centered as it includes proactive outreach to promote self-management and treatment adherence, and to engage patients in coordination of services.^23^ Additionally, the team involved in this model is a multidisciplinary team of health care professionals who are empowered to work at the top of their professional training.^23^

**Assignment:**

*After you finish reading the information above, please begin working on the following assignment. The expectation is to complete this assignment by the end of this rotation* [*or learning experience*]*. As part of this activity, please review the most recent published literature to ensure understanding of the definition of integrated behavioral health models and related terminology, such as team-driven care, population-based care/population management, integrated care. This literature review will help you answer the assignment questions.*

Based on your observations during this rotation and what you have learned about different models of integrated behavioral health:

Mid rotation:

1. Please discuss some of the advantages and limitations of the current practice of integrated BH at [*the name of the rotation site*].
2. Which model(s) of care is (are) being practiced at [*the name of the rotation site*]? Provide evidence to support your answer.

At the end of the rotation:

1. Imagine you are the psychiatrist [*or the BH provider*] conducting the BH services at [*the name of the rotation site*]. Would you consider making changes to the current practice?
2. If yes to #3, please discuss some of the advantages and barriers to the change(s) you are considering.

**References**

1. World Health Organization. Integrated care models: an overview. *Geneva World Health Organ*. Published online 2016.

2. Shaw S, Rosen R, Rumbold B. What is integrated care. *Lond Nuffield Trust*. 2011;7:1-23.

3. Integrated Care. American Psychiatric Association. Accessed May 24, 2022. https://psychiatry.org:443/psychiatrists/practice/professional-interests/integrated-care

4. Minkman MM. The current state of integrated care: an overview. *J Integr Care*. Published online 2012.

5. Kwan BM, Nease DE. The State of the Evidence for Integrated Behavioral Health in Primary Care. In: Talen MR, Burke Valeras A, eds. *Integrated Behavioral Health in Primary Care*. Springer New York; 2013:65-98. doi:10.1007/978-1-4614-6889-9_5

6. Coates D, Coppleson D, Schmied V. Integrated physical and mental healthcare: an overview of models and their evaluation findings. *JBI Evid Implement*. 2020;18(1):38-57.

7. Rodgers M, Dalton J, Harden M, Street A, Parker G, Eastwood A. Integrated Care to Address the Physical Health Needs of People with Severe Mental Illness: A Mapping Review of the Recent Evidence on Barriers, Facilitators and Evaluations. *Int J Integr Care*. 18(1):9. doi:10.5334/ijic.2605

8. Yeung CHT, Santesso N, Zeraatkar D, et al. Integrated multidisciplinary care for the management of chronic conditions in adults: an overview of reviews and an example of using indirect evidence to inform clinical practice recommendations in the field of rare diseases. *Haemophilia*. 2016;22:41-50.

9. Gerrity M. Evolving models of behavioral health integration: Evidence update 2010–2015. *N Y NY Milbank Meml Fund*. Published online 2016.

10. Woltmann E, Grogan-Kaylor A, Perron B, Georges H, Kilbourne AM, Bauer MS. Comparative effectiveness of collaborative chronic care models for mental health conditions across primary, specialty, and behavioral health care settings: systematic review and meta-analysis. *Am J Psychiatry*. 2012;169(8):790-804. doi:10.1176/appi.ajp.2012.11111616

11. Valentijn PP, Boesveld IC, van der Klauw DM, et al. Towards a taxonomy for integrated care: a mixed-methods study. *Int J Integr Care*. 2015;15:e003.

12. National Survey on Drug USe and Health. 2020 National Survey of Drug Use and Health (NSDUH) Releases | CBHSQ Data. Published 2020. Accessed February 4, 2022. https://www.samhsa.gov/data/release/2020-national-survey-drug-use-and-health-nsduh-releases

13. National Alliance on Mental Health. Mental Health by the Numbers. Published online 2022. https://www.nami.org/mhstats

14. Canady VA. SAMHSA NSDUH report finds increases in mental health, co-occurring disorders. *Ment Health Wkly*. 2020;30(36):1-3.

15. National Institutes of Mental Health. Mental Illness. National Institute of Mental Health (NIMH). Accessed February 4, 2022. https://www.nimh.nih.gov/health/statistics/mental-illness

16. Butler M, Kane RL, McAlpine D, et al. Integration of mental health/substance abuse and primary care. *Database Abstr Rev Eff DARE Qual-Assess Rev Internet*. Published online 2008.

17. Butler M, Kane RL, McAlpine D, et al. Does integrated care improve treatment for depression?: a systematic review. *J Ambulatory Care Manage*. 2011;34(2):113-125.

18. Raney L. Integrated Care: The Evolving Role of Psychiatry in the Era of Health Care Reform. *Psychiatr Serv*. 2013;64(11):1076-1078. doi:10.1176/appi.ps.201300311

19. Heath B, Wise Romero P, Reynolds K. A standard framework for levels of integrated healthcare. *Wash DC SAMHSA-HRSA Cent Integr Health Solut*. Published online 2013.

20. Boudreau DM, Capoccia KL, Sullivan SD, et al. Collaborative care model to improve outcomes in major depression. *Ann Pharmacother*. 2002;36(4):585-591.

21. Finley PR, Rens HR, Pont JT, et al. Impact of a collaborative care model on depression in a primary care setting: a randomized controlled trial. *Pharmacother J Hum Pharmacol Drug Ther*. 2003;23(9):1175-1185.

22. Unützer J, Harbin H, Schoenbaum M, Druss B. The collaborative care model: An approach for integrating physical and mental health care in Medicaid health homes. *Health Home Inf Resour Cent*. Published online 2013:1-13.

23. Collaborative / Integrated Care. Accessed January 8, 2024. https://www.psychiatry.org:443/psychiatrists/practice/professional-interests/integrated-care
