# Supplementary material for: Integrated Behavioral Health: A Curriculum for Residents in Rural and Community Psychiatry
Source: MedEdPORTAL. 2024 Dec 20;20:11468. doi: 10.15766/mep_2374-8265.11468 (PMC11659397; doi:10.15766/mep_2374-8265.11468)
Supplement: Supplementary file 1 — Background for Facilitators.docxLearner Guide.docxSession 1 Facilitator Guide.docxSession 2 Facilitator Guide.docxSession 3 Facilitator Guide.docxSession 4 Facilitator Guide.docxFacilitator Guide Slides.pptxSimulation Scenario.docxEvaluation Survey.docx [file mep_2374-8265.11468-s001.zip › C. Session 1 Facilitator Guide.docx]

**Appendix C**

**Session 1 Facilitator Guide**

This document includes three main sections related to the first session: 1. Orientation, 2. Learning Activities, and 3. Debriefing and Mutual Feedback. The Orientation section provides guidance for introducing the learning goal and general structure of the rotation to the learner. The learning Activities section focuses on Session 1 activities and includes detailed teaching instructions on components of integrated care. The session ends with debriefing and the exchange of feedback, as outlined in the third section. The quotation marks indicate the talking points that the facilitator may use for communication with the learner.

**Orientation:**

- **Goal Statement**

“The learning goal that we have in mind for you is to acquire and apply knowledge relevant to integrated behavioral health. We have defined two learning objectives toward that goal: 1- To compare different models of behavioral health (BH) integration; 2- To critically appraise clinical practice, using your knowledge of different models of BH integration.”

*Notes:*

*Although not essential to implementation and outcome of this curriculum, it is generally advised to inquire about the learner’s learning goals. To identify the learner’s goal(s), you may ask them “Are there any goals that you wish to accomplish in addition to acquiring and applying knowledge relevant to integrated behavioral health?”*

*Also, it is important to clearly communicate expectations and details related to exchange of formative feedback, including timing, frequency, and aims, at the beginning of a learning activity. To do so, you may say, “I give you feedback at the end of each session/day on areas in which you did well and areas to improve. I would like to receive feedback from you at the end of each session on what went well and things I could change to improve the learners’ experience.”*

*Specifically, in clinical rotations, it is crucial to provide orientation to the physical space and introduce to colleagues and staff. For example, you may say, “At this point, I will orient you to the clinic space and introduce you to clinic staff and other providers.”*

- **Structure of the Rotation**

The following table offers a general overview of the rotation structure, which you may review with the learner in session 1. This table is also included in the Learner Guide that you email to the learner before the end of Session 1.

| **Time** | **Activities** |
| --- | --- |
| Session 1 | Discussion of learning goal, objectives, and activities.  Observation of the clinical practice.  Participation in and discussion of the components of integrated care. |
| Session 2 | Completion of the assignment’s questions 1 and 2.  Observation of the clinical practice.  Participation in and discussion of the components of integrated care. |
| Session 3 | Discussion of the assignment’s questions 1 and 2.  Observation of the clinical practice.  Participation in and discussion of the components of integrated care. |
| Session 4 | Completion of the assignment’s questions 3 and 4.  Discussion of the assignment’s questions 3 and 4.  Observation of the clinical practice.  Participation in and discussion of the components of integrated care.  Reflection on the entire learning experience. |

It is of note that this curriculum can be completed in two sessions instead of four as outlined in the following table:

| **Time** | **Activities** |
| --- | --- |
| Session 1 | Discussion of learning goal, objectives, and activities.  Observation of the clinical practice.  Participation in and discussion of the components of integrated care.  Completion of the assignment questions 1 and 2.  Discussion of the assignment questions 1 and 2. |
| Session 2 | Observation of the clinical practice.  Participation in and discussion of the components of integrated care.  Completion of the assignment questions 3 and 4.  Discussion of the assignment questions 3 and 4.  Reflection on the entire learning experience. |

**Learning Activities:**

The table below provides Session 1 learning activities and the associated talking points for the facilitator. You may use the Facilitator Talking Points to explain the learning activities to the learner.

Following the table, you will find the teaching instructions for components of integrated care that you will focus on during Session 1 and the following sessions (E-consults, curbside consults, warm handoffs).

| **Learning Activities** | **Facilitator Talking Points** |
| --- | --- |
| Discussion of learning objectives and activities. | “I have emailed/will email you a document (Learner Guide) that contains an overview of integrated BH and an assignment, along with the learning goal and objectives that we just discussed.  The assignment will guide your focus during this rotation and provides the context for discussion of the details related to integrated care.  You are expected to respond to the first two assignment prompts by the second session (mid-rotation) and the last two prompts by the fourth session (end-of- rotation).  You will obtain the information needed for completion of the assignment by shadowing and observing me in the clinic and completing a literature review. You can either email me your responses to the assignment, or we can discuss them verbally.” |
| Observation of the clinical practice. | “While you shadow me in the clinic, please pay attention and try to distinguish various ways in which I engage in patient care. These include direct patient care, curbside consults, warm handoffs, E-consults, etc. You will use these observations to respond to the rotation’s assignment.” |
| Participation in and discussion of the components of integrated care. | “When I receive communication from primary care providers (e.g. curbside consults, warm handoffs, E-consults, etc), I will include you in the exchange so that you can participate in providing integrated services. I will provide direct supervision and support while you engage in those clinical services. This opportunity will allow you to observe my role and activities in the clinic and gain first-hand experience as an integrated provider.  If we do not get any E-consults during your rotation, I will email you an example of an E-consult case. You should then reply to me as if you are writing to the primary care provider.” |

- **Teaching Instructions for Components of Integrated Care:**

E-Consults:

Please ensure that the learners are familiar with the definition of E-consults. As described in Appendix A, an E-consult is “an asynchronous communication between healthcare providers that occurs within a shared electronic health record (EHR) or secure Web-based platform.”

Below are two E-consult cases that you may use in case you do not receive any E-consults during a learner’s rotation. You may email the learner one of the cases and ask them to respond as if they are replying to a primary care provider (PCP)’s message on the patient’s electronic medical record.

Case #1-

- PCP: A 58-year-old woman with depression, anxiety, insomnia, no medical conditions, and normal TSH. An outside provider tried quetiapine, which made her groggy. She experienced dizziness on gabapentin 100mg. I started her on fluoxetine 20mg less than two weeks ago. Today she came back for follow up and reported no improvement of her symptoms. Her appetite is poor and she has lost weight. What would you recommend?
- Consultant: Thanks for consulting me on this case. In my opinion there are two different ways to approach this case:

Approach #1: Because it has been less than two weeks since the patient was started on fluoxetine, it would make sense to wait for at least two more weeks before considering switching to a different antidepressant, unless she has side effects from the fluoxetine. Some of the common side effects of fluoxetine include nausea, diarrhea, increased anxiety, and insomnia. It is notable that most of these side effects resolve with continued medication use.

Approach #2: Considering the patient’s poor appetite, weight loss, and insomnia, which are most likely secondary to depression and anxiety (assuming potential medical etiologies such as malignancies, autoimmune disorders, and obstructive sleep apnea have been ruled out), it would be appropriate to switch from fluoxetine to mirtazapine. Mirtazapine would help with depression and anxiety as well as poor appetite, weight loss, and insomnia. You may start at 15mg at bedtime and increase to 30mg in 1-2 weeks based on response and tolerability. Monitor for common side effects, including xerostomia and constipation. Other side effects such as drowsiness and increased appetite would likely benefit this patient.

I hope this is helpful. Please let me know if you have any questions.

Case #2-

- PCP: A 45-year-old man with depression and generalized anxiety disorder on vilazodone that was started by an outside provider. Insurance no longer covers vilazodone. What alternative medication would you recommend? Medical history is positive for HTN, chronic pain, vasculitis, Wegener’s disease, vitamin D deficiency.
- Consultant: Thanks for consulting me on this case. Vilazodone is a serotonin partial agonist and reuptake inhibitor with minimal or no effect on reuptake of norepinephrine or dopamine. Therefore, its mechanism of action differs to some extent from SSRIs and SNRIs.

Considering the patient’s chronic pain, it would be appropriate to try an SNRI (venlafaxine or duloxetine) if he has not tried either of these medications and if his blood pressure is well controlled (SNRIs can increase blood pressure, so monitoring is necessary.)

Regarding vitamin D deficiency, the patient would benefit from education on the role of vitamin D deficiency in depression and the importance of treatment adherence to ensure adequate treatment.

Regarding autoimmune diseases (vasculitis, Wegener’s disease), chronic inflammation may lead to depression. Therefore, effective treatment of the autoimmune diseases is important in managing depression. Some research studies have shown that N-Acetyl Cysteine helps treat depression in patients with autoimmune disease. If the patient is interested in trying this medication, we can provide instructions on how to take it.

If the patient has already tried and failed SNRIs, details of past medication trials are necessary before making other recommendations. In that case, the patient might benefit from further assessment by one of our psychiatrists.

Curbside Consults:

Please ensure that the learners are familiar with the definition of curbside consults. As described in Appendix A, a curbside consult is “an informal process whereby a physician obtains information or advice from another physician to assist in the management of a particular patient. The consultant is generally unfamiliar with the patient and has not reviewed the patient's chart or examined the patient.”

Warm Handoff:

Please ensure that the learners are familiar with the definition of warm handoffs. As described in Appendix A, a warm handoff is “a handoff that is conducted in person, between two members of the health care team, in front of the patient (and family if present).”

**Debriefing and Mutual Feedback:**

You may use the following talking points to debrief this session and exchange feedback with the learner:

“How was your day at the clinic today? Was there anything particularly challenging or noteworthy that you would like to discuss? What worked well for you today? What do you think we could improve to enhance your experience during this rotation? I would also like to give you feedback based on my observation of your strengths and areas for improvement. Would that be ok?”
