# Supplementary material for: Integrated Behavioral Health: A Curriculum for Residents in Rural and Community Psychiatry
Source: MedEdPORTAL. 2024 Dec 20;20:11468. doi: 10.15766/mep_2374-8265.11468 (PMC11659397; doi:10.15766/mep_2374-8265.11468)
Supplement: Supplementary file 1 — Background for Facilitators.docxLearner Guide.docxSession 1 Facilitator Guide.docxSession 2 Facilitator Guide.docxSession 3 Facilitator Guide.docxSession 4 Facilitator Guide.docxFacilitator Guide Slides.pptxSimulation Scenario.docxEvaluation Survey.docx [file mep_2374-8265.11468-s001.zip › D. Session 2 Facilitator Guide.docx]

**Appendix D**

**Session 2 Facilitator Guide**

This document includes two sections related to the second session: 1. Learning Activities, and 2. Debriefing and Mutual Feedback. You can review the details of the teaching instructions for the components of integrated care (curbside consults, warm handoffs, and E-consults) in the Session 1 Facilitator Guide.

**Learning Activities:**

The table below provides Session 2 learning activities and their associated talking points for the facilitator. You can use the Facilitator Talking Points included in this table to explain the learning activities to the learner.

| **Learning Activities** | **Facilitator Talking Points** |
| --- | --- |
| Observation of the clinical practice. | “Similar to Session 1, while you shadow me in the clinic, please observe the components of integrated care such as direct care, curbside consults, warm handoffs, and E-consults. I want to ensure that you are able to identify and differentiate these components.” |
| Participation in and discussion of the components of integrated care. | “Similar to Session 1, I will include you in curbside consults, warm handoffs, and E-consults, while providing direct supervision and support.”  *Notes for the facilitator:*   - *Please ensure that the learner completes at least one E-consult, whether from a PCP or one of the provided examples, before the end of Session 2.* - *Please consider sharing documents such as your referral guidelines and any workflows you have generated for your clinic with the learner.* |
| Completion of the assignment’s questions 1 and 2. | “I would like you to complete the first two assignment questions before the end of today:   1. Please discuss some of the advantages and limitations of the current practice of integrated BH at [*the name of the rotation site*]. 2. Which model(s) of care is (are) being practiced at [*the name of the rotation site*]? Provide evidence to support your answer.   You may start by re-reading the overview of BH integration provided in the Learner Guide. Additionally, please review some of the most recent research publications. It is acceptable to skip some of today’s clinical activities to focus on completing this task.” |

**Debriefing and Mutual Feedback:**

You may use the following talking points to debrief this session and exchange feedback with the learner:

“How was your day at the clinic today? Was there anything particularly challenging or noteworthy that you would like to discuss? What worked well for you today? What do you think we could improve to enhance your experience during this rotation? I would also like to give you feedback based on my observation of your strengths and areas for improvement. Would that be ok?”
