# Supplementary material for: Integrated Behavioral Health: A Curriculum for Residents in Rural and Community Psychiatry
Source: MedEdPORTAL. 2024 Dec 20;20:11468. doi: 10.15766/mep_2374-8265.11468 (PMC11659397; doi:10.15766/mep_2374-8265.11468)
Supplement: Supplementary file 1 — Background for Facilitators.docxLearner Guide.docxSession 1 Facilitator Guide.docxSession 2 Facilitator Guide.docxSession 3 Facilitator Guide.docxSession 4 Facilitator Guide.docxFacilitator Guide Slides.pptxSimulation Scenario.docxEvaluation Survey.docx [file mep_2374-8265.11468-s001.zip › E. Session 3 Facilitator Guide.docx]

**Appendix E**

**Session 3 Facilitator Guide**

This document includes two sections related to the third session: 1. Learning Activities, and 2. Debriefing and Mutual Feedback.

**Learning Activities:**

The table below provides Session 3 learning activities and their associated talking points for the facilitator. You may use the Facilitator Talking Points included in this table to explain the learning activities to the learner.

Following the table, you will find teaching instructions for the assignment questions 1 and 2. Completion of these two questions and the related teaching points is the most important learning activity for this session.

| **Learning Activities** | **Facilitator Talking Points** |
| --- | --- |
| Observation of the clinical practice. | “Similar to the last two sessions, you will shadow me in the clinic today to observe and distinguish different components of integrated care, such as direct care, curbside consults, warm handoffs, and E-consults.” |
| Participation in and discussion of the components of integrated care. | “Similar to the last two sessions, you will have the opportunity to participate in curbside consults, warm handoffs, and E-consults today.” |
| Discussion of the assignment’s questions 1 and 2. | “We will discuss questions 1 and 2 of the assignment before the end of today. This will provide the opportunity to explore different models of integrated care and allow me to offer targeted teaching on those models. You may email me your responses to those assignment questions. If you choose to do so, we can discuss your responses while I review your email. Otherwise, we will talk about them, and you can refer to any notes you took while answering the questions.” |

- **Teaching instructions for assignment questions 1 and 2:**

1. **Please discuss some of the advantages and limitations of the current practice of integrated BH at [*the name of the rotation site*].**

Below are the advantages of integrated BH in general. Some or all of these advantages may apply to any specific rotation site for this curriculum.

- Improves quality of care.
- Improves population health.
- Improves access to care (serves a higher volume of patients).
- Improves outcome of care for patients with multiple comorbidities.
- Reduces cost of care.
- Provides person-centered care.
- Promotes a biopsychosocial approach to care.
- Provides team-based care.
- Provides measurement-based care (uses valid and reliable tools to monitor and track symptoms).
- Provides evidence-based care.
- Provides context for both individual-based and population-based treatments.
- Reduces stigma.
- Reduces cultural barriers to behavioral health care.
- Increases patient satisfaction.
- Increases provider satisfaction.
- Lowers the no-show rate.
- Lowers wait times.
- Provides context for warm handoffs for BH providers to triage patients and make recommendations.
- PCPs reported that it frees up their time.
- Patients receive BH care in a familiar environment.
- Reduces referrals to specialty care clinics, thus reducing the burden on those clinics.
- Enhances adherence to care for patients who may not follow through with referrals to specialty care clinics.

Below are the limitations of integrated BH in general. One or both of these limitations, along with the lack of some of the above-mentioned advantages, may apply to any specific rotation site for this curriculum.

- Cannot provide the intensive care required for severe and persistent mental illnesses and high-risk patients.
- Cannot provide long-term care.

The goal of this exercise is for the learner to recognize some of the advantages and limitations of the integrated model that you have implemented at your practice site. This recognition will be based on their observation of your practice of integrated care in contrast with other models described in the literature, as well as the traditional models of outpatient care. Certain aspects of integrated care that would be considered strengths and advantages might be missing from a particular practice site and therefore identified as limitations. For example, at our practice site, the lack of a care registry (and thus population health care), the absence of screening and enhanced case finding, and the lack of structured team meetings have been identified as limitations by the residents. This exercise prepares the learners for the last two assignment questions.

1. **Which model(s) of care is (are) being practiced at [*the name of the rotation site*]? Provide evidence to support your answer.**

This exercise prompts learners to observe and examine your integrated services and identify your model(s) of integration. It offers an opportunity to review different models of integrated care with the learners. You may utilize Appendix A to conduct this discussion. If the learner has correctly identified your practice’s model, ensure that they are able to articulate the rationale for ruling out other models and choosing a particular model.

For example, at UNM SRMC Family Practice clinic, we have developed integrated services that are a hybrid of two models of integration (co-located and BH consultant models) to improve quality of and access to care for the underserved patient population of the clinic within the existing resources and the preexisting structure of the family practice clinic. The residents usually notice an agreed-upon referral guideline, the PCP and BH providers co-location, and a shared electronic medical record. They also observe that, for some patients, the psychiatrist provides direct patient care and eventually refers them back to PCP. Accordingly, they identify the co-location component of the services.

Moreover, the residents observe single-point entry, team-based, measurement-based, evidence-based, and accountable care, an agreed-upon warm handoff workflow along with warm handoffs, curbside consults, and E-consults as components of integrated care. These, in addition to the shared location and EMR, cue them to the psychiatrist’s role as a BH consultant. They usually recognize the elements and components associated with this model and describe it as an integrated model without necessarily knowing its specific name. This has offered an opportunity to provide further teaching and resources.

Sometimes, the presence of components such as team-based, measurement-based, evidence-based, and accountable care leads the residents to the idea that our implemented model is the Collaborative Care Model (CoCM). This provides a great opportunity to review population-based care and highlight the care manager and care registry as key components of the CoCM model. If a learner does not bring up the CoCM in their response to this question, prompts such as “what is your understanding of the CoCM? Why do you think our practice is not CoCM?” help ensure their understanding of this model. The same strategy may be used to confirm the learner’s comprehension of other models, such as coordinated and collocated care models.

**Debriefing and Mutual Feedback:**

You may use the following talking points to debrief this session and exchange feedback with the learner:

“How was your day at the clinic today? Was there anything particularly challenging or noteworthy that you would like to discuss? What worked well for you today? What do you think we could improve to enhance your experience during this rotation? I would also like to give you feedback based on my observation of your strengths and areas for improvement. Would that be ok?”
