# Supplementary material for: Integrated Behavioral Health: A Curriculum for Residents in Rural and Community Psychiatry
Source: MedEdPORTAL. 2024 Dec 20;20:11468. doi: 10.15766/mep_2374-8265.11468 (PMC11659397; doi:10.15766/mep_2374-8265.11468)
Supplement: Supplementary file 1 — Background for Facilitators.docxLearner Guide.docxSession 1 Facilitator Guide.docxSession 2 Facilitator Guide.docxSession 3 Facilitator Guide.docxSession 4 Facilitator Guide.docxFacilitator Guide Slides.pptxSimulation Scenario.docxEvaluation Survey.docx [file mep_2374-8265.11468-s001.zip › F. Session 4 Facilitator Guide.docx]

**Appendix F**

**Session 4 Facilitator Guide**

This document includes two sections related to the fourth session: 1. Learning Activities, and 2. Debriefing and Mutual Feedback.

**Learning Activities:**

The table below provides Session 4 learning activities and their associated talking points for the facilitator. You may use the Facilitator Talking Points included in this table to explain the learning activities to the learner.

Following the table, you will find teaching instructions for the assignment questions 3 and 4, as well as the final learning activity of this rotation, which is the learner’s reflection. Please prioritize completing and discussing assignment questions 3 and 4 before the end of this session.

| **Learning Activities** | **Facilitator Talking Points** |
| --- | --- |
| Observation of the clinical practice.  Participation in and discussion of the components of integrated care. | “Similar to previous sessions, you may shadow me in the clinic today to observe and participate in curbside consults, warm handoffs, and E-consults. However, today’s priority is to complete questions 3 and 4 of the assignment. If you prefer, you can start by working on those, and once you finish, join me in the clinical activities.” |
| Completion of the assignment’s questions 3 and 4. | “I would like you to complete assignment questions 3 and 4 before the end of today:   1. Imagine you are the psychiatrist [*or the BH provider*] conducting the BH services at [*the name of the rotation site*]. Would you consider making changes to the current practice? 2. If yes to #3, please discuss some of the advantages and barriers to the change(s) you are considering.   Your observations of the clinical practice at this rotation site and the literature you have reviewed on different models of integrated BH, including the elements and components described at different practices, will help you answer these questions.” |
| Discussion of the assignment’s questions 3 and 4. | “At some point before the end of today, we will discuss questions 3 and 4 of the assignment. I look forward to hearing your thoughts and ideas because they can help improve our practice. Additionally, they could potentially be the focus of Quality Improvement projects for you or other residents.  Similar to last time, you may email me your responses to those assignment questions. If you choose to do so, we can discuss your responses while I review your email. Otherwise, we will talk about them, and you can refer to any notes you took while answering the questions.” |
| Reflection on the learning experience during this rotation. | “This is the end of your last day on this rotation. I would like you to take a few minutes to reflect on your experience during this rotation and answer the following questions:   - How did this experience influence your view of outpatient behavioral health practices and/or your interests? - What are you interested in learning next?   This will help consolidate your learning and potentially assist us in improving this curriculum. It could also help identify components for a follow-up rotation on integrated behavioral health later in the residency program.” |

- **Teaching instructions for assignment questions 3 and 4:**

1. **Imagine you are the psychiatrist [*or the BH provider*] conducting the BH services at [*the name of the rotation site*]. Would you consider making changes to the current practice?**

The learners respond to this and the following question based on their assessment of your integrated services compared to different models and practices presented in the literature or observed elsewhere. Their responses can vary due to different factors such as the depth of their literature review on integrated care, as well as their creativity and critical thinking. Reassure the learners that their ideas can help improve the practice and will not be taken as criticism.

For example, some of the residents who rotated through our clinic suggested the following changes:

- Educational sessions for PCPs on differentiating chronic from acute psychiatric conditions.
- Hiring extra BH workforce.
- Addition of multidisciplinary team meetings.
- Addition of a care registry and a care manager.
- Addition of brief psychotherapy interventions.
- Implementation of active depression screening for the primary care clinic’s patient population.
- Implementation of active screening of patients susceptible to mental illness.

1. **If yes to #3, please discuss some of the advantages and barriers to the change(s) you are considering.**

Examples of advantages of the changes suggested in question 3:

- Improving efficiency with existing resources.
- Improving quality of care.
- Improving patient safety.
- Promoting population health.
- Promoting proactive approach to care.
- Enhancing the biopsychosocial approach to care.

Examples of barriers to the changes suggested in question 3:

- The absence of a budget or incentives, such as challenges of funding a full-time care manager or the cost of embedding and utilizing a care registry.
- Lack of mechanisms for reimbursement for some proposed BH services.
- Reimbursement being dependent on individual provider billing.
- **Teaching instructions for the learner’s reflection:**

The Learner Reflection was created as the rotation’s final learning activity to help consolidate learning, with the understanding that it could also be potentially utilized for assessing the curriculum and planning curricula for advanced residency years. Below are the questions and a summary of example responses from the residents who completed our rotation.

- **How did this experience influence your view of outpatient behavioral health practices and/or your interests?**

Summary of example responses:

One resident said that their perception of integrated psychiatry changed significantly during this rotation. They explained that prior to this experience, they did not see as much value in integrated psychiatry compared to specialty care clinics. Another resident expressed a positive surprise, stating they liked integrated psychiatry more than they had expected.

- **What are you interested in learning next?**

Summary of example responses:

Some residents expressed interest in further exploring integrated psychiatry during their elective rotations to learn the specifics of conducting integrated services and decide whether to consider it for their future job.

**Debriefing and Mutual Feedback:**

You may use the following talking points to debrief this session and exchange feedback with the learner:

“How was your day at the clinic today? Was there anything particularly challenging or noteworthy that you would like to discuss? What worked well for you today? What do you think we could improve to enhance your experience during this rotation? I would also like to give you feedback based on my observation of your strengths and areas for improvement. Would that be ok?”
