# Supplementary material for: Integrated Behavioral Health: A Curriculum for Residents in Rural and Community Psychiatry
Source: MedEdPORTAL. 2024 Dec 20;20:11468. doi: 10.15766/mep_2374-8265.11468 (PMC11659397; doi:10.15766/mep_2374-8265.11468)
Supplement: Supplementary file 1 — Background for Facilitators.docxLearner Guide.docxSession 1 Facilitator Guide.docxSession 2 Facilitator Guide.docxSession 3 Facilitator Guide.docxSession 4 Facilitator Guide.docxFacilitator Guide Slides.pptxSimulation Scenario.docxEvaluation Survey.docx [file mep_2374-8265.11468-s001.zip › G. Facilitator Guide Slides.pptx]

## Slide 1
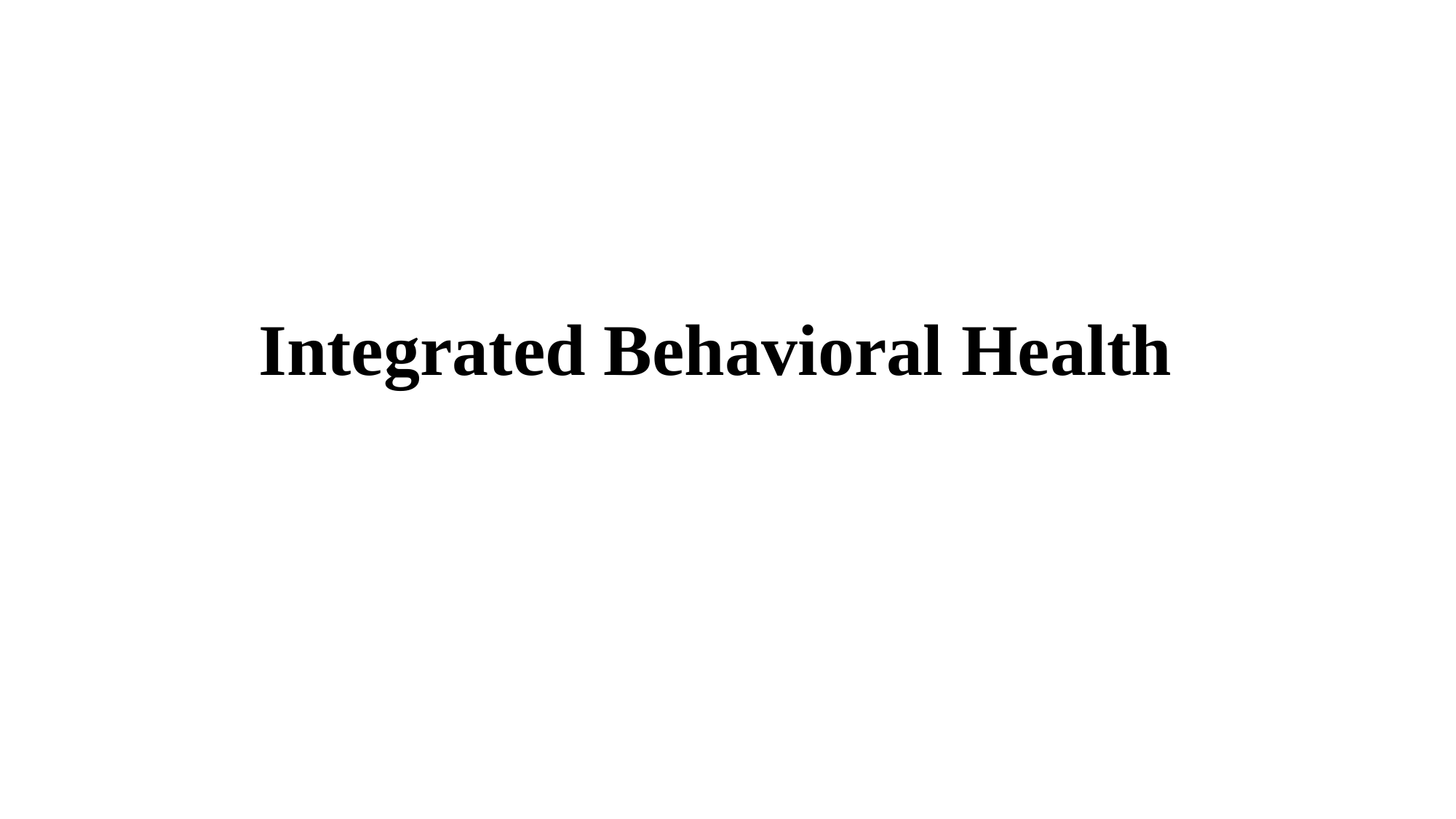

# Integrated Behavioral Health

## Slide 2
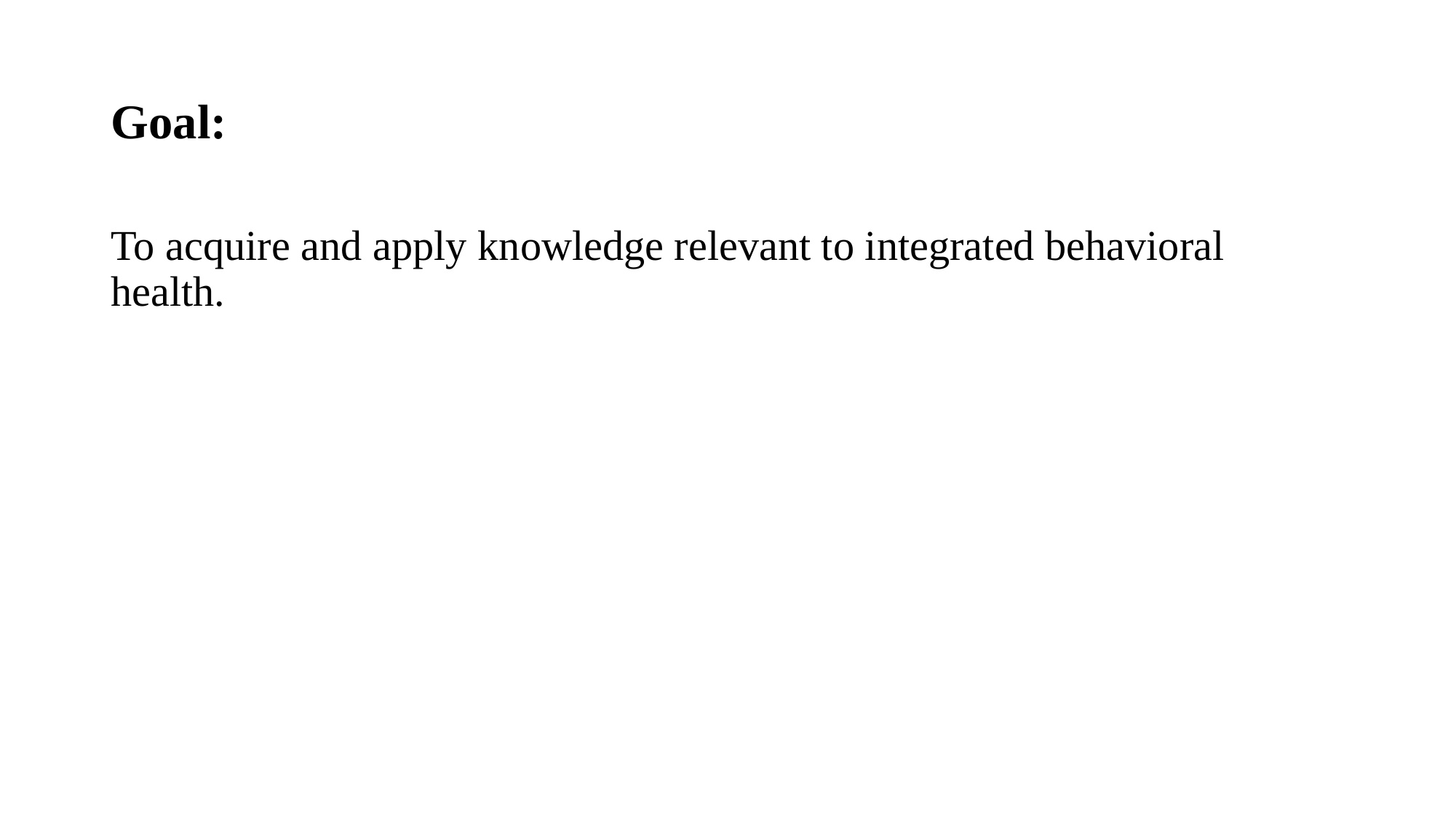

# Goal:
To acquire and apply knowledge relevant to integrated behavioral health.

## Slide 3
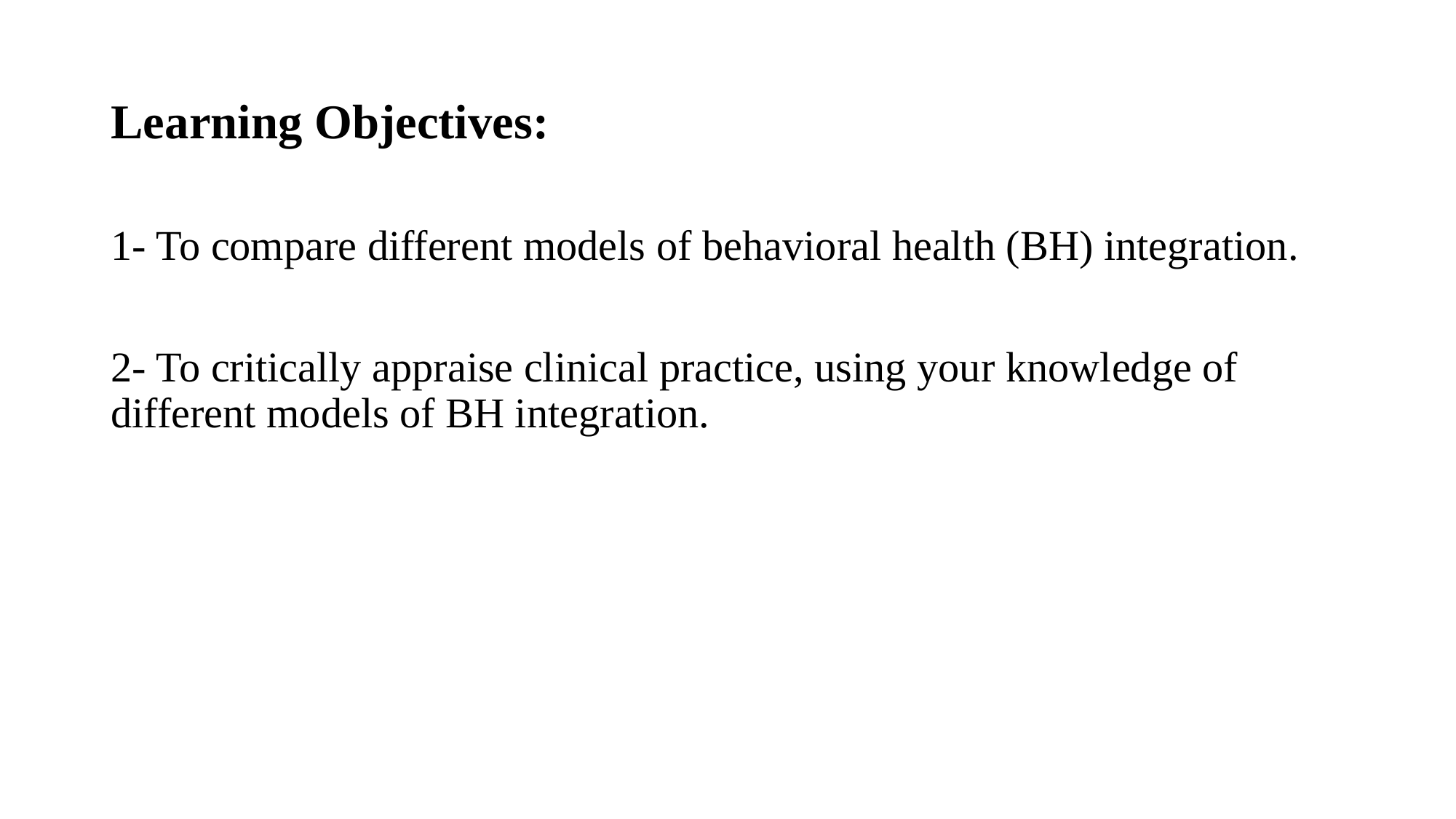

# Learning Objectives:
1- To compare different models of behavioral health (BH) integration.
2- To critically appraise clinical practice, using your knowledge of different models of BH integration.

## Slide 4
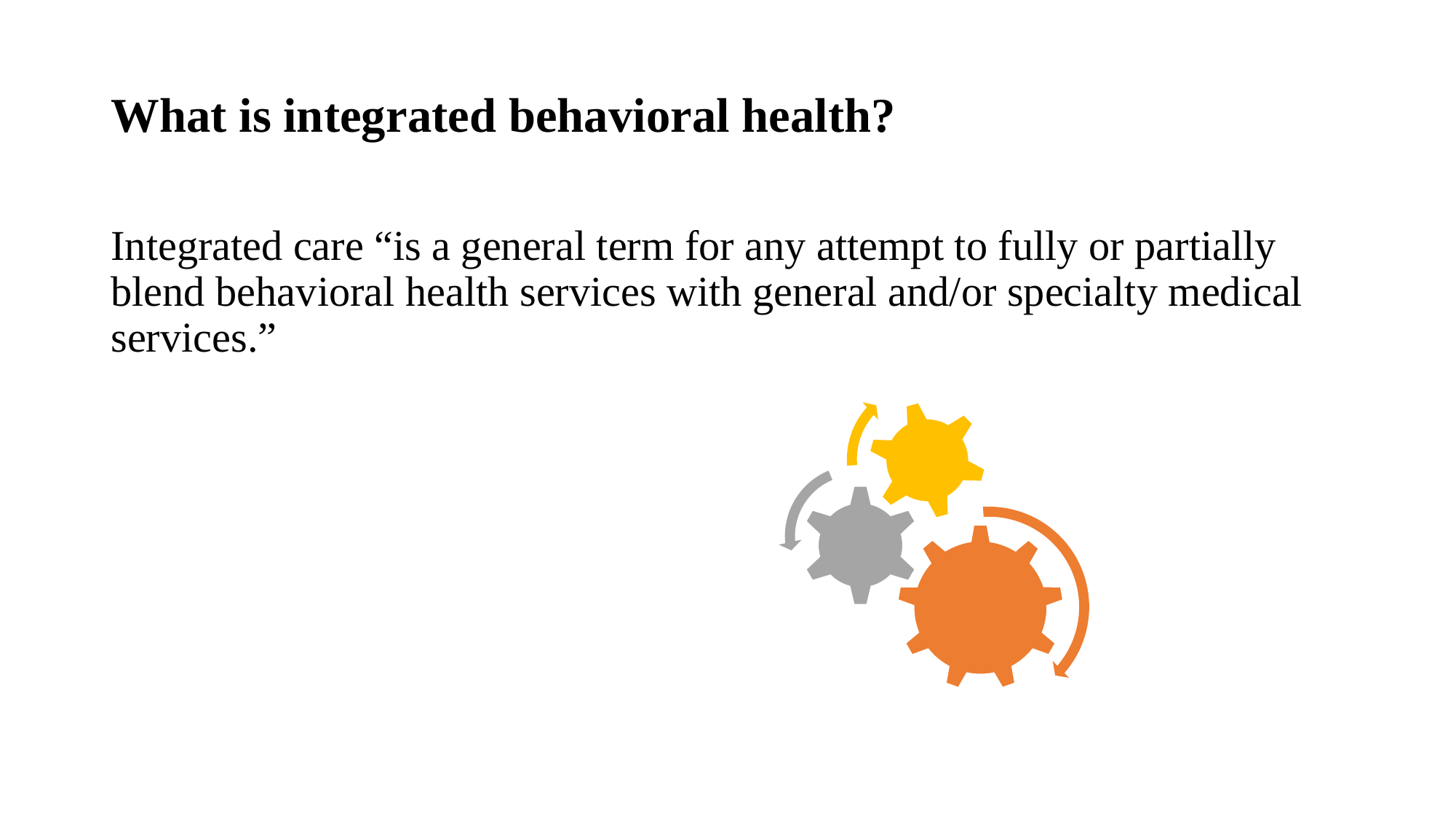

# What is integrated behavioral health?
Integrated care “is a general term for any attempt to fully or partially blend behavioral health services with general and/or specialty medical services.”

## Slide 5
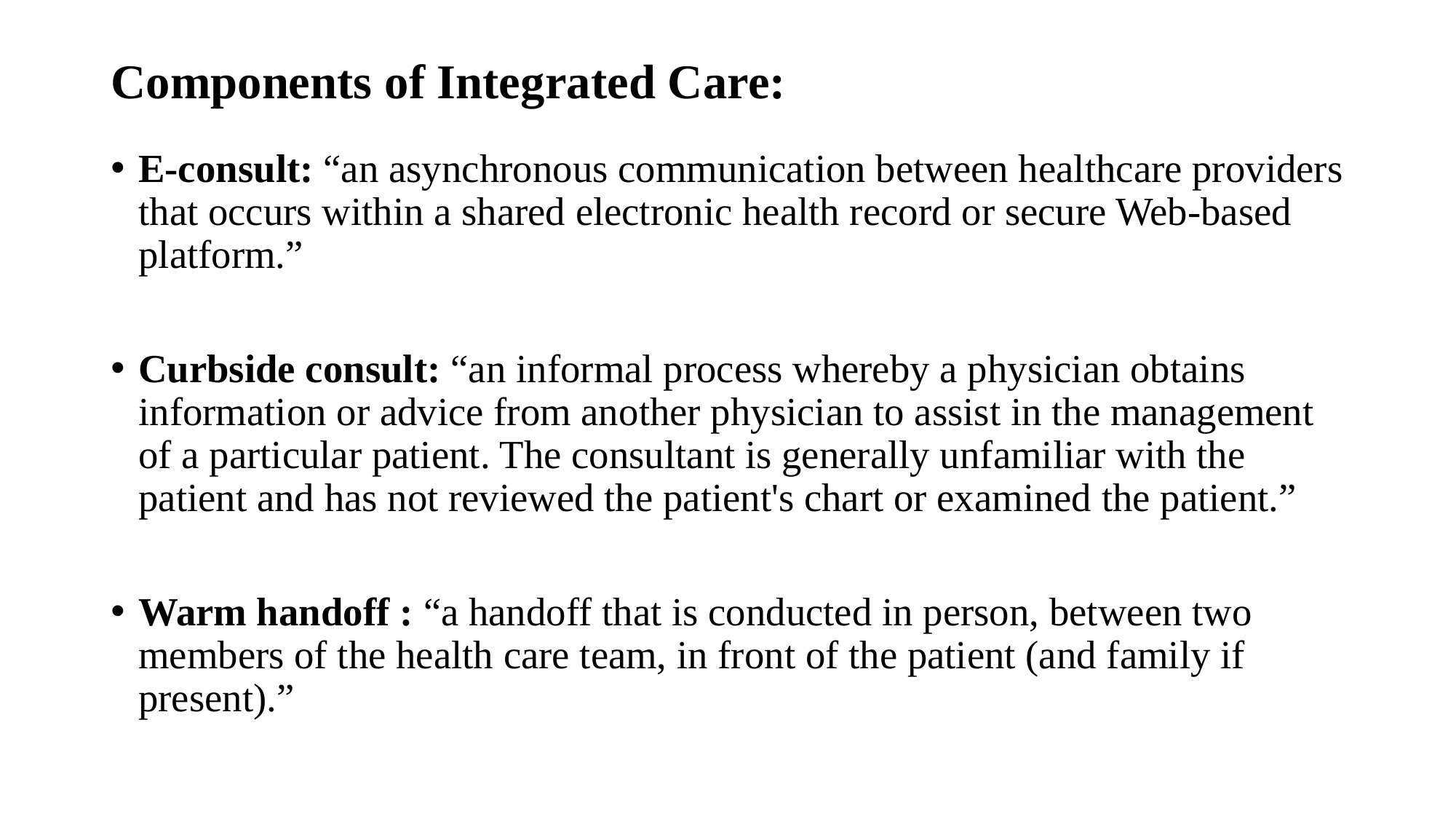

# Components of Integrated Care:
E-consult: “an asynchronous communication between healthcare providers that occurs within a shared electronic health record or secure Web-based platform.”
Curbside consult: “an informal process whereby a physician obtains information or advice from another physician to assist in the management of a particular patient. The consultant is generally unfamiliar with the patient and has not reviewed the patient's chart or examined the patient.”
Warm handoff : “a handoff that is conducted in person, between two members of the health care team, in front of the patient (and family if present).”

## Slide 6
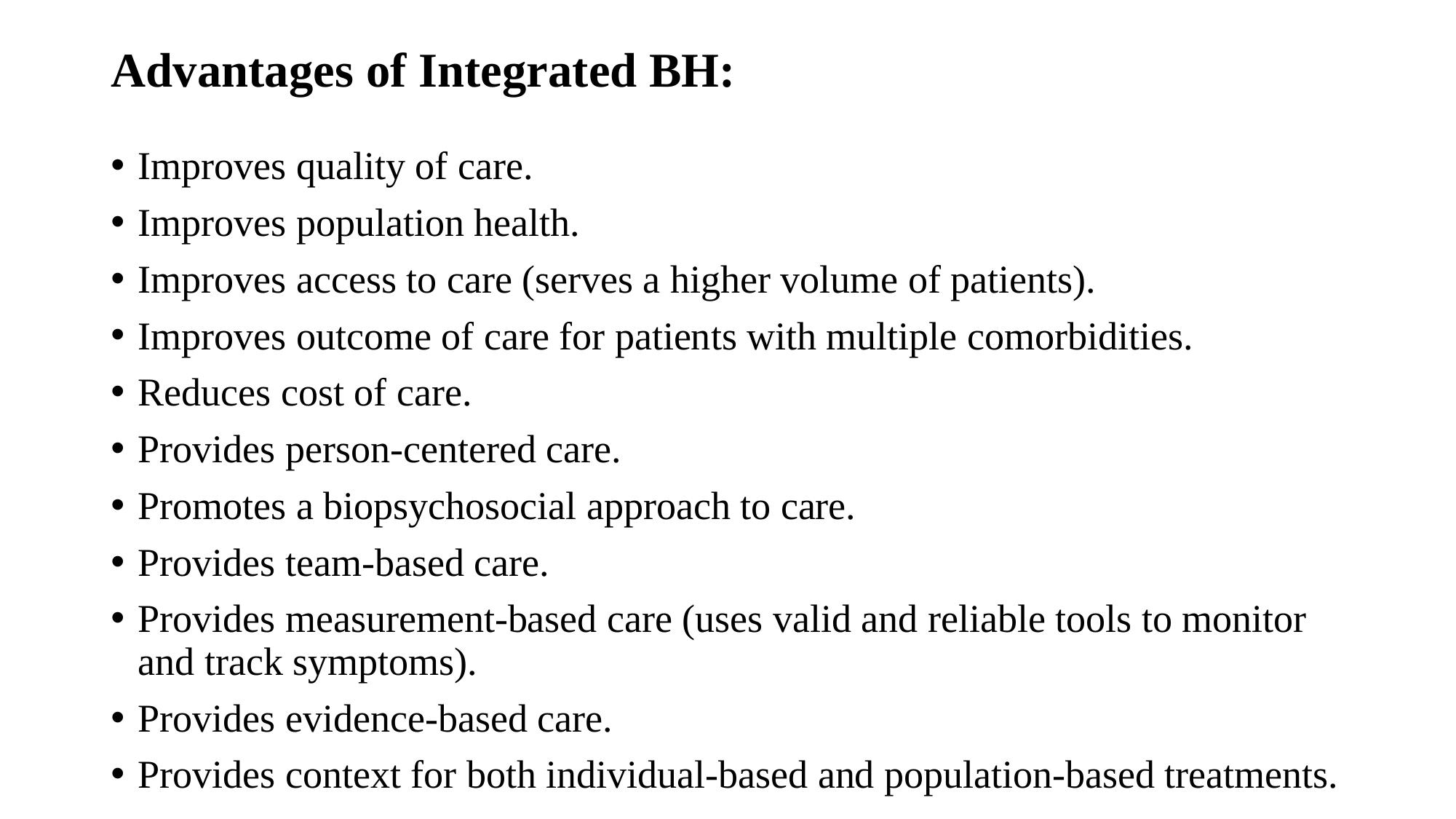

# Advantages of Integrated BH:
Improves quality of care.
Improves population health.
Improves access to care (serves a higher volume of patients).
Improves outcome of care for patients with multiple comorbidities.
Reduces cost of care.
Provides person-centered care.
Promotes a biopsychosocial approach to care.
Provides team-based care.
Provides measurement-based care (uses valid and reliable tools to monitor and track symptoms).
Provides evidence-based care.
Provides context for both individual-based and population-based treatments.

## Slide 7
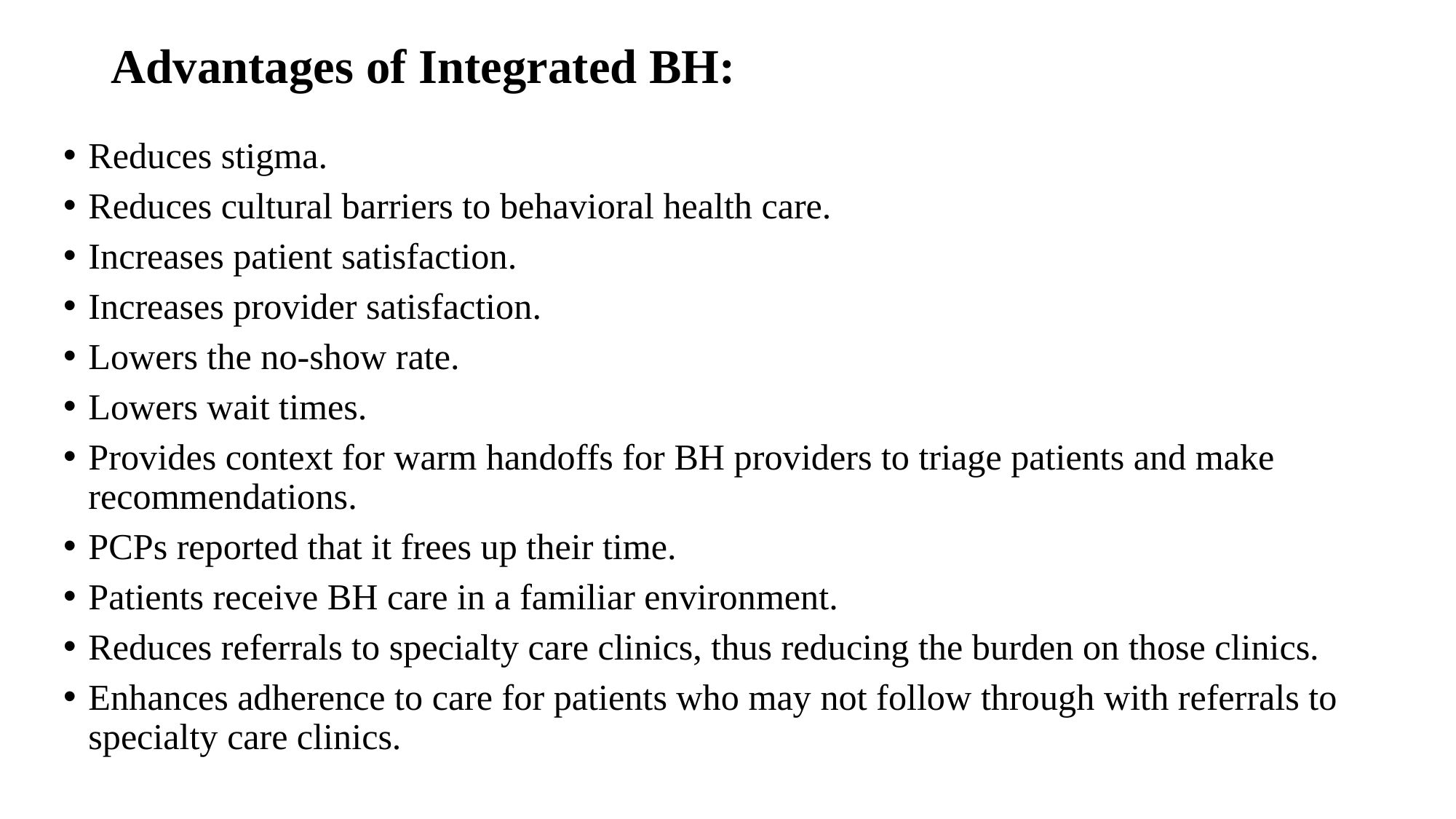

# Advantages of Integrated BH:
Reduces stigma.
Reduces cultural barriers to behavioral health care.
Increases patient satisfaction.
Increases provider satisfaction.
Lowers the no-show rate.
Lowers wait times.
Provides context for warm handoffs for BH providers to triage patients and make recommendations.
PCPs reported that it frees up their time.
Patients receive BH care in a familiar environment.
Reduces referrals to specialty care clinics, thus reducing the burden on those clinics.
Enhances adherence to care for patients who may not follow through with referrals to specialty care clinics.

## Slide 8
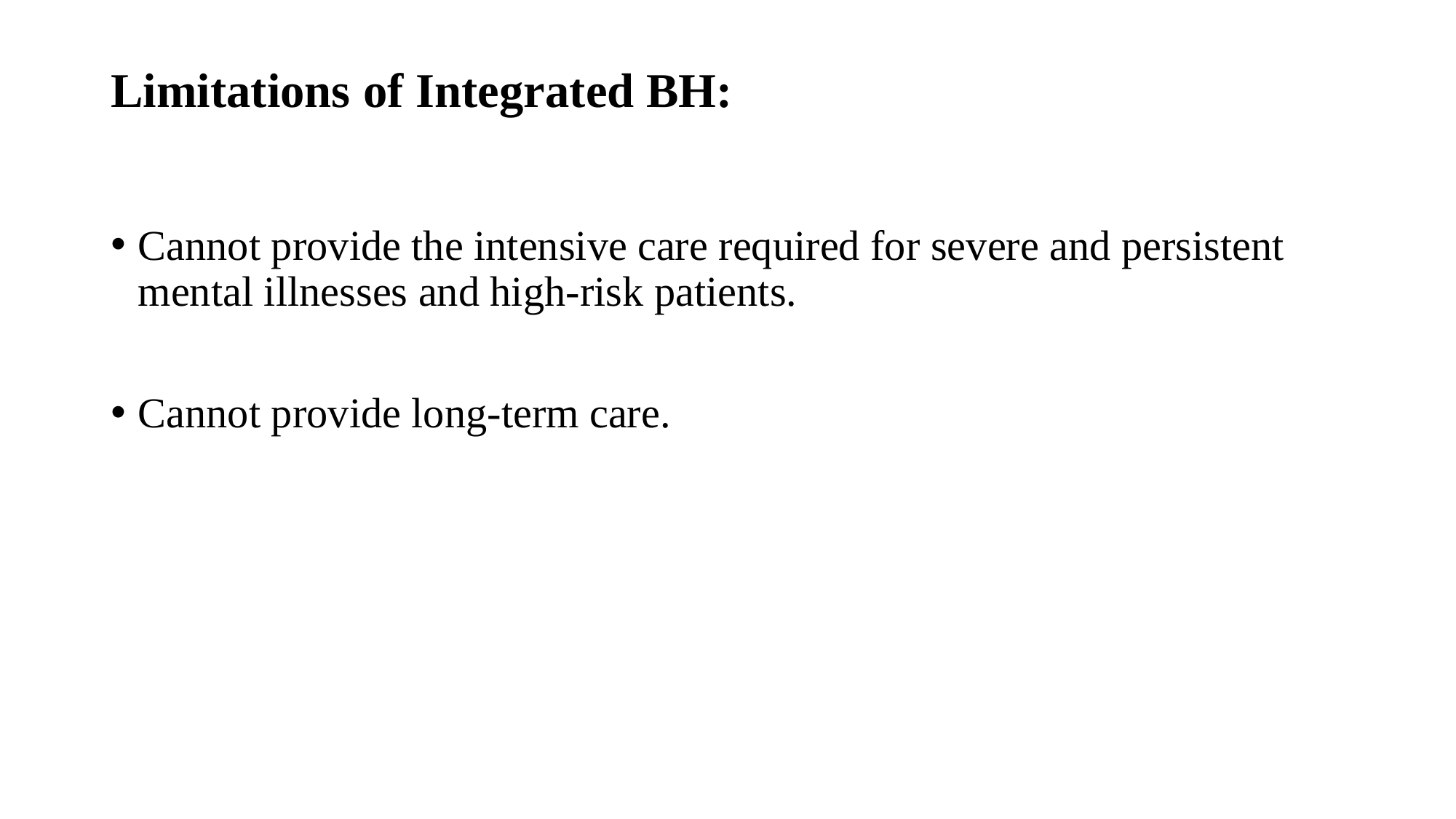

# Limitations of Integrated BH:
Cannot provide the intensive care required for severe and persistent mental illnesses and high-risk patients.
Cannot provide long-term care.

## Slide 9
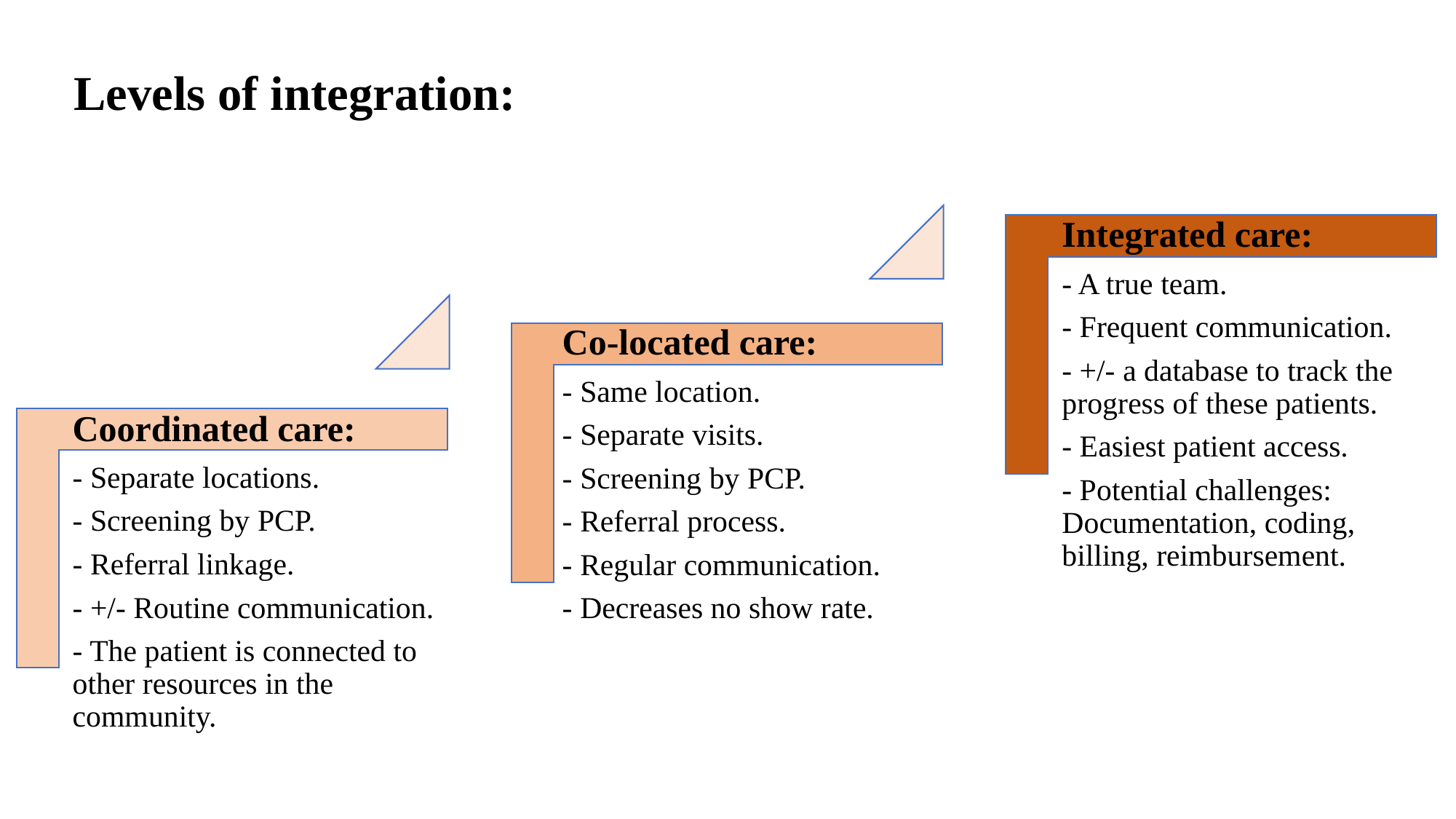

# Levels of integration:

## Slide 10
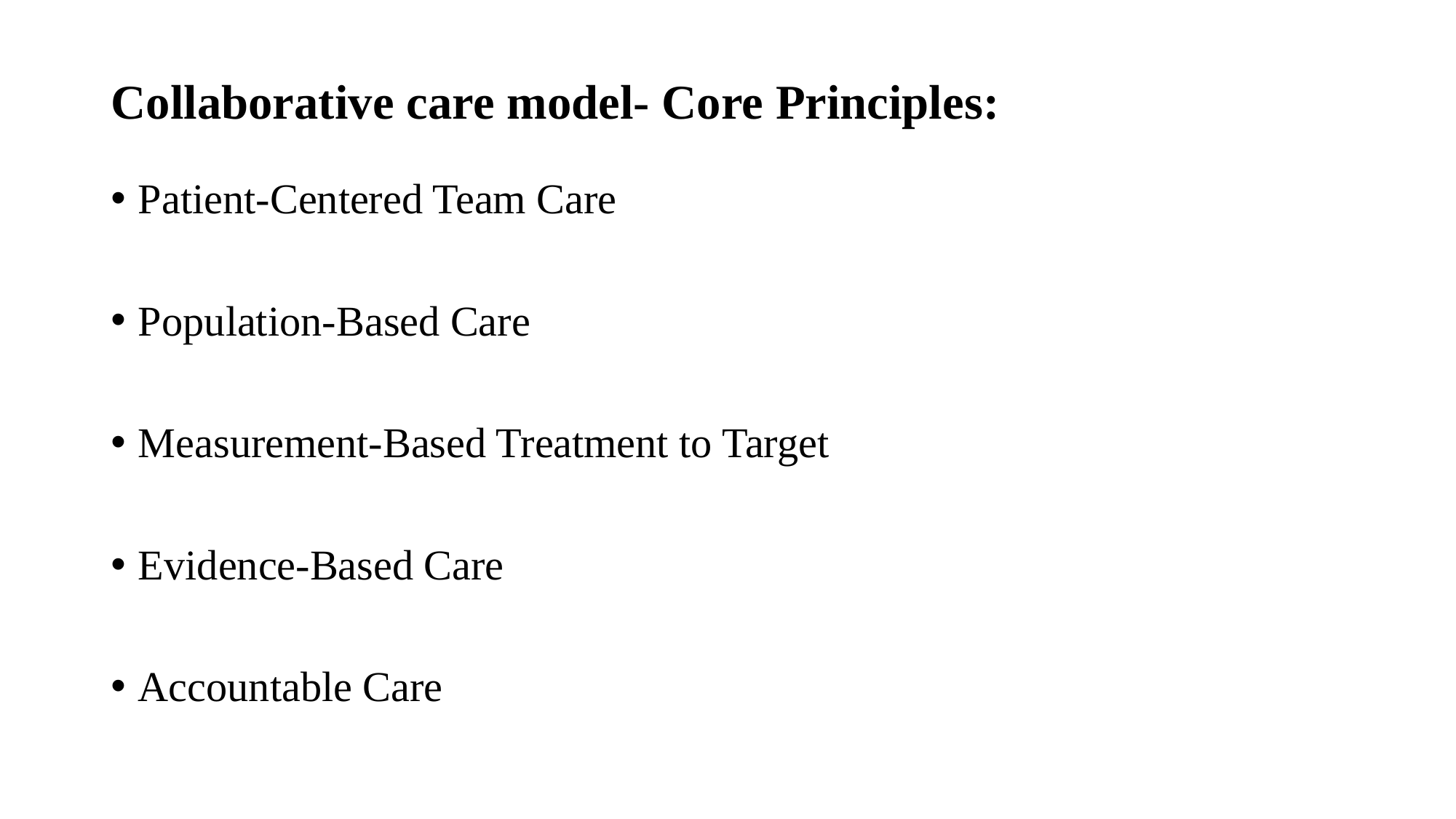

# Collaborative care model- Core Principles:
Patient-Centered Team Care
Population-Based Care
Measurement-Based Treatment to Target
Evidence-Based Care
Accountable Care

## Slide 11
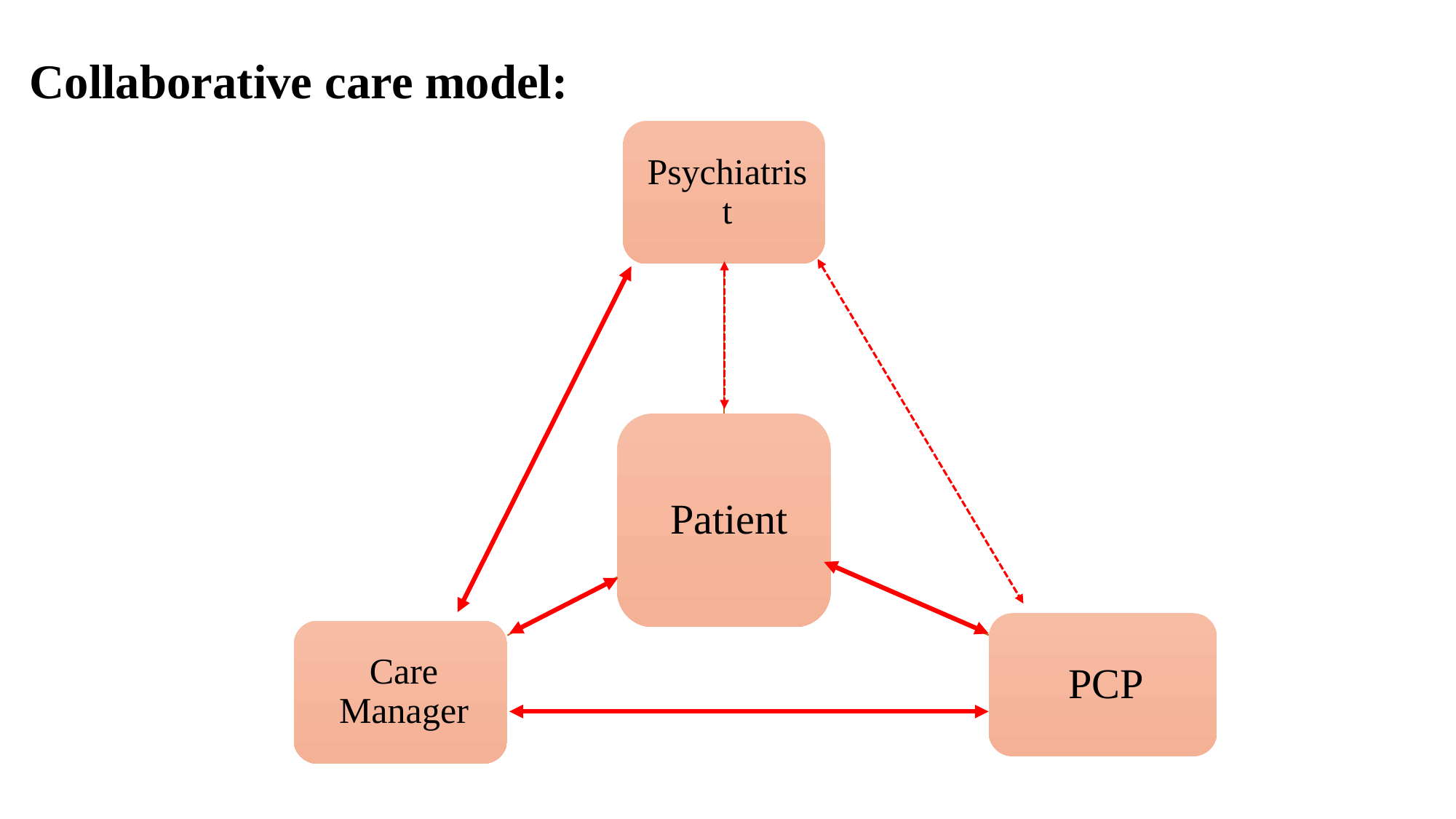

# Collaborative care model:

## Slide 12
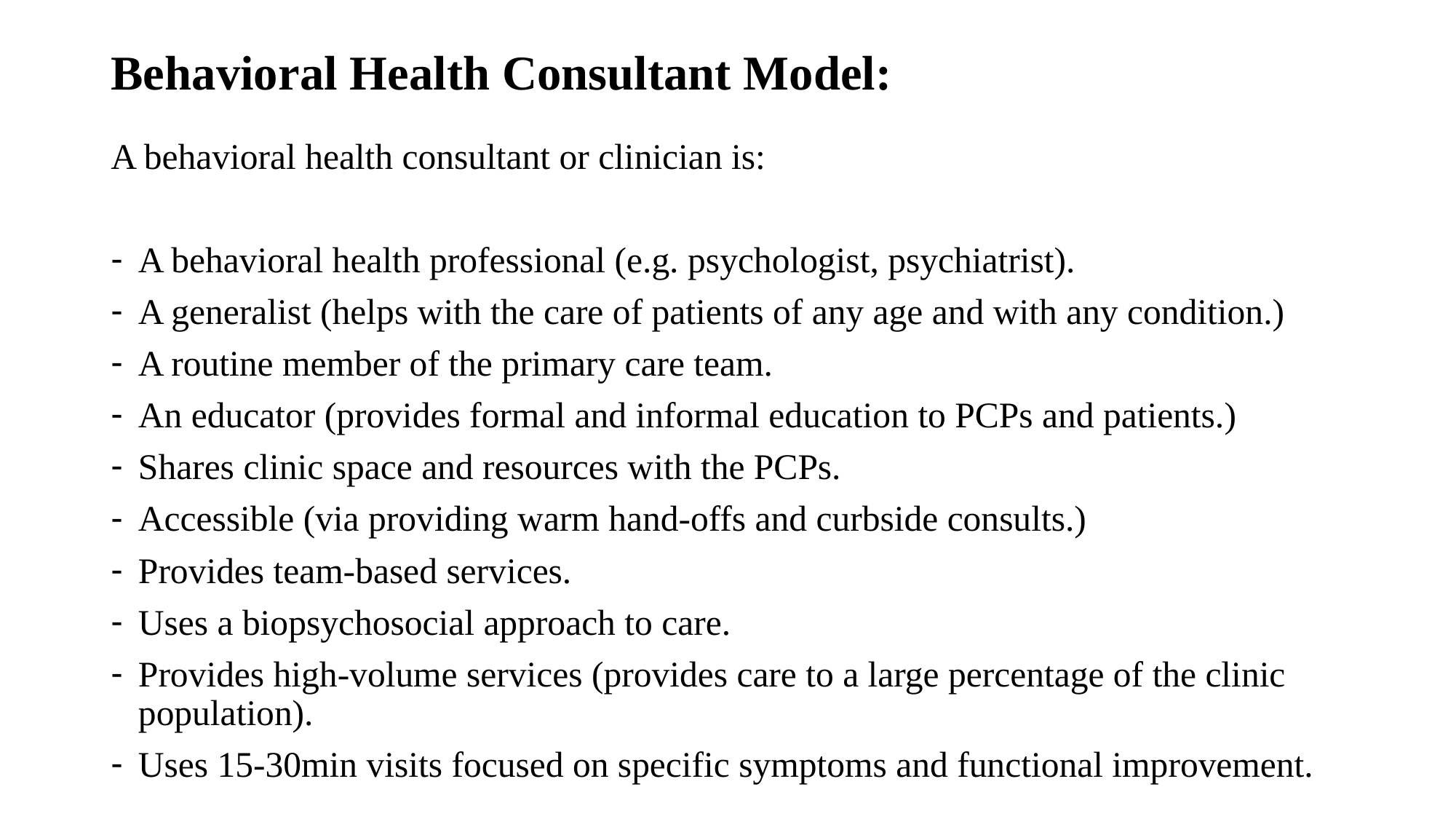

# Behavioral Health Consultant Model:
A behavioral health consultant or clinician is:
A behavioral health professional (e.g. psychologist, psychiatrist).
A generalist (helps with the care of patients of any age and with any condition.)
A routine member of the primary care team.
An educator (provides formal and informal education to PCPs and patients.)
Shares clinic space and resources with the PCPs.
Accessible (via providing warm hand-offs and curbside consults.)
Provides team-based services.
Uses a biopsychosocial approach to care.
Provides high-volume services (provides care to a large percentage of the clinic population).
Uses 15-30min visits focused on specific symptoms and functional improvement.
