# Supplementary material for: Integrated Behavioral Health: A Curriculum for Residents in Rural and Community Psychiatry
Source: MedEdPORTAL. 2024 Dec 20;20:11468. doi: 10.15766/mep_2374-8265.11468 (PMC11659397; doi:10.15766/mep_2374-8265.11468)
Supplement: Supplementary file 1 — Background for Facilitators.docxLearner Guide.docxSession 1 Facilitator Guide.docxSession 2 Facilitator Guide.docxSession 3 Facilitator Guide.docxSession 4 Facilitator Guide.docxFacilitator Guide Slides.pptxSimulation Scenario.docxEvaluation Survey.docx [file mep_2374-8265.11468-s001.zip › H. Simulation Scenario.docx]

**Appendix G**

**Simulation Scenario**

This document includes a Simulation Scenario, the associated assignment questions, and the teaching instructions for those questions. If you do not have a pre-existing clinical rotation for integrated care, you may use this Simulation Scenario instead of Appendices C, D, E, and F. Plan to complete this activity in two sessions.

Facilitators can use Appendix A (Background for Facilitators) and Appendix B (Learner Guide) to prepare for teaching and conducting discussions on the assignment questions.

Learners should review the Learner Guide (Appendix B) first, then read the Simulation Scenario and answer the assignment questions in the order presented in this document. As part of this activity, learners should also examine the most recent literature on behavioral health (BH) integration models, including definitions of terminology (e.g., team-driven care, population-based care/population management, and integrated care.) This literature review, combined with the content of the Learner Guide, will help them answer the assignment questions.

The following table provides an overview of this activity’s structure and estimated time, which you should review with the learner in session 1. Keep in mind that each learner’s background, such as level of familiarity with systems of care, preparedness, and interest in this topic, might impact the time they spend completing the assignment questions and engaging in the discussions with the facilitator.

| **Time** | **Activities** |
| --- | --- |
| Session 1  (95-125min) | Discuss learning objectives and activities (5min).  Review Simulation Scenario Day 1 (10min).  Complete assignment questions 1 and 2 (60-80min).  Discuss assignment questions 1 and 2 (20-30min). |
| Session 2  (80-90min) | Review Simulation Scenario Day 2 (5min).  Complete an E-consult scenario (15min).  Discuss an E-consult (10min).  Complete assignment questions 3 and 4 (30min).  Discuss assignment questions 3 and 4 (20-30min). |

**Simulation Scenario:**

Today is your day 1 of two days at a Family Medicine (FM) clinic in an underserved area, shadowing Dr. A, a behavioral health (BH) provider, who is a generalist. Please read the description of your observations and answer the assignment questions.

**Day 1-**

Dr. A works at the FM clinic three days a week. While Dr. A orients you to the clinic space and introduces you to staff and providers, Dr. B, one of the primary care providers (PCPs), shares that prior to Dr. A joining this clinic, patients experienced very long wait times to see a behavioral health (BH) specialist.

Dr. A then provides information on the clinic’s background, explaining that at the beginning of the integrated services, several patients were referred for severe depression and lack of response to various antidepressants. These patients met the criteria for bipolar disorder and responded well to the treatment of bipolar depression. This created the opportunity to discuss with the PCPs the importance of ruling out bipolar disorder in patients presenting with depression.

At 9 AM, Dr. A starts seeing patients, including one new patient and two follow-up visits, in the exam rooms at the FM clinic. In between patients, Dr. A explains to you that these patients were referred by the clinic’s PCPs based on a referral guideline. Dr. A created this guideline while establishing the integrated care services, and it was approved by the clinic leadership and providers.

You also observe that all of Dr. A’s patients complete PHQ-9 (Patient Health Questionnaire) and GAD-7 (Generalized Anxiety Disorder) questionnaires. Dr. A carefully reviews the results and explains to the patients that these questionnaires help screen for and track depression (PHQ-9) and anxiety (GAD-7). Dr. A identifies shared treatment goals, including the ideal PHQ-9 and GAD-7 scores, points out the changes in the scores when patients return for follow-up, and reviews the justification for and the evidence supporting treatment recommendations for individual patients.

Later in the morning, you notice that one of the follow-up patients has reached and remained in remission for the last six months. You observe that Dr. A offers to refer this patient back to the PCP. Dr. A explains to the patient that the PCP will continue to manage their care and can refer them to Dr. A again if needed in the future. Dr. A also mentions that this would enable Dr. A to continue to accept more patients for BH care. The patient feels comfortable with this plan and expresses gratitude for the BH care they received. Dr. A then updates the patient’s PCP via a message on the electronic medical record (EMR) and ensures that the PCP has no questions or concerns regarding this plan.

Additionally, you notice that, in between patients, Dr. A uses the same workspace as the PCPs to complete documentation and other tasks related to patient care.

At this point, Dr. B walks out of an exam room to the providers’ workstation and asks Dr. A for help with a patient who is in the clinic for a follow-up regarding high blood pressure. Dr. B shares the patient’s pertinent history and explains that the patient is reporting problems with anxiety and insomnia. Dr. B is concerned that the anxiety and insomnia might be contributing to the patient’s high blood pressure. You all walk to the exam room, where Dr. B introduces you and Dr. A to the patient. Dr. A spends 25 minutes with the patient, initiates treatment, and offers a follow-up appointment, which the patient accepts.

Returning to the workstation, Dr. A explains to you that this was a warm handoff, for which Dr. A has created a specific workflow. You notice a copy of the warm handoff workflow, which includes a flow chart detailing the steps for PCPs and the clinic medical assistants in the warm handoff process. These steps include establishing a new patient encounter number to ensure reimbursement for Dr. A’s services.

In the afternoon, Dr. A sees another new patient and a few follow-up patients. One patient, in particular, is appreciative of receiving BH care in the FM clinic and says it is helpful to sit in the lobby of the FM clinic without anyone knowing the reason for their visit. The patient reports initial anxiety about seeing a BH provider; however, coming to a familiar clinic space and staff helped to overcome that discomfort.

In between patients, Dr. A checks their EMR messages. Among them is a message from one of the clinic’s PCPs. It includes information about a patient’s history and a question regarding the best next step. Dr. A responds to the message with recommendations and explains to you that this is an E-consult.

Later in the afternoon, one of Dr. A’s patients with comorbid major depressive disorder, post-traumatic stress disorder, and borderline personality disorder presents with worsened depression and suicidal ideations. This is partly due to recent psychosocial and financial stressors. Dr. A completes an assessment for safety and ensures that the patient is not at imminent risk for suicide. Dr. A adjusts the patient’s treatment and asks the clinic’s social worker to help the patient access resources in the community, as well as provide ongoing assessment, interventions, and support between appointments. The social worker and the patient agree on a plan for regular telephonic follow-up until the patient’s next appointment with Dr. A. Additionally, Dr. A initiates an EMR message thread including the patient’s PCP and the social worker so they can collaborate and coordinate care through this critical phase of the patient’s care.

In the afternoon, Dr. C, a PCP, approaches Dr. A with a question about a patient that Dr. C is about to see. Dr. C and Dr. A exchange information, and Dr. A gives some recommendations for the appropriate next steps. Dr. A explains to you that this is a curbside consult.

Assignment:

Answer the following questions using the description of this clinic’s Day1 and what you have learned about different models and practices of BH integration from the content of the Learner Guide and your literature review.

1. Please discuss some of the advantages and limitations of the integrated BH practice described.
2. Which model(s) of Primary Care-BH are being practiced at this Family Medicine clinic? Provide evidence to support your answer.

**Day 2-**

On Day 2, you observe that, similar to Day 1, Dr. A has new and follow-up patients in the morning and afternoon.

Around 4 PM, one of the clinic nurses notifies Dr. A that the daughter of a clinic patient is on the phone. She reports that her 75-year-old father, who has multiple medical conditions including a history of diabetes and several strokes, appears confused and seems to be talking to people who are not there. Her father lives alone, and she lives in a different state. She regularly talks to him on the phone and recently noticed that he was sounding increasingly confused. Therefore, she flew here to check on him. Dr. A reviews the patient’s EMR and notices that the patient has not been seen in this clinic or other parts of the healthcare system for over six months. Dr. A recommends that the patient be evaluated in the emergency department right away.

Before the end of the day, Dr. A shares with you an E-consult from one of the PCPs and asks you to help generate a response.

Assignment:

Answer the following questions using the description of this clinic’s Day1 and Day2, as well as what you have learned about different models and practices of BH integration from the content of the Learner Guide and your literature review.

1. Imagine you are the provider or a member of the team conducting the integrated BH services at this Family Practice clinic. Would you consider making changes to the current practice?
2. If yes to #3, please discuss some of the advantages and barriers to the change(s) you are considering.

**Teaching Instructions for the assignment questions:**

1. **Please discuss some of the advantages and limitations of the integrated BH practice described.**

The goal of this exercise is for the learner to recognize some of the advantages and limitations of the described integrated BH practice in contrast with other models of integrated care described in the literature, as well as the traditional models of outpatient psychiatric care.

The advantages of the described practice based on Day 1 observations include:

- Improvement in quality of care.
- Improvement in access to care (serves a higher volume of patients).
- Improvement in outcome of care for patients with multiple comorbidities.
- Reduction in cost of care.
- Providing person-centered care.
- Promoting biopsychosocial approach to care.
- Providing team-based care.
- Providing measurement-based care (uses valid and reliable tools to monitor and track symptoms).
- Providing evidence-based care.
- Patients receive BH care in a familiar environment.
- Reducing stigma.
- Reducing cultural barriers to behavioral health care.
- Increasing patient satisfaction.
- Increasing provider satisfaction.
- Lowering the no-show rate.
- Lowering wait times.
- Providing context for warm handoffs for BH providers to triage patients and make recommendations.
- Freeing up PCPs’ time.
- Reducing referrals to specialty care clinics, thus reducing the burden on those clinics.
- Enhancing adherence to care for patients who may not follow through with referrals to specialty care clinics.

Some of the other advantages of BH integration that do not apply to the practice described in this scenario are:

- Improvement in population health.
- Providing context for both individual-based and population-based treatments.

The limitations of this practice based on Day 1 observations and the nature of integrated psychiatry in general include:

- Inability to provide intensive care required for severe and persistent mental illnesses and high-risk patients.
- Inability to provide long-term care.

Some of the other limitations of this practice compared to other models and practices of integrated care include:

- Lack of integration of billing and reimbursement.
- Lack of a population health approach.
- Lack of screening and enhanced case finding.
- Lack of structured team meetings.

1. **Which model(s) of Primary Care-BH are being practiced at this Family Medicine clinic? Provide evidence to support your answer.**

This exercise prompts the learner to identify the model(s) of integration at the described practice. It offers an opportunity to review different models of integrated care with the learners. The correct answer and the rationale are discussed below. You may also utilize Appendix A to conduct this discussion. If the learner has correctly identified your practice’s model, ensure that they are able to articulate the rationale for ruling out other models and choosing a particular model.

The described practice is a hybrid of two models of integration (co-located and BH consultant models). Some of the elements of integrated care described in the scenario include an agreed-upon referral guideline, the co-location of PCP and BH providers, and a shared EMR. Additionally, the BH provider delivers direct patient care to some of the patients while eventually referring them back to PCPs. These are key elements of the co-located aspect of these integrated services.

Moreover, single-point entry, team-based, measurement-based, evidence-based, and accountable care, an agreed-upon warm handoff workflow, along with warm handoffs, curbside consults, and E-consults are components specific to a higher level of integration. These, in addition to the shared location and EMR, are components of the BH consultant model. It is noteworthy that learners might recognize the components associated with BH consultant model and describe it as an integrated model without knowing its specific name. This presents an opportunity to offer further teaching and resources on this model.

Sometimes, the presence of components such as team-based, measurement-based, evidence-based, and accountable care can lead the learners to the idea that the described practice is the Collaborative Care Model (CoCM). This provides a great opportunity to review population-based care and highlight the care manager and care registry as key components of the CoCM model. If a learner does not bring up the CoCM in their response to this question, prompts such as “what is your understanding of the CoCM? Why do you think our practice is not CoCM?” help ensure their understanding of this model. The same strategy may be used to confirm the learner’s comprehension of other models such coordinated and collocated care models.

1. **Imagine you are the provider or a member of the team conducting the integrated BH services at this Family Practice clinic. Would you consider making changes to the current practice?**

The learners will answer this and the following question based on their assessment of the described integrated practice compared to different models and practices presented in the literature observed elsewhere. Their responses can vary due to different factors such as the depth of their literature review on integrated care, as well as their creativity and critical thinking.

Some of the potential changes that the learners may suggest based on what they notice missing from the described practice include:

- Educational sessions for PCPs on differentiating chronic from acute psychiatric conditions.
- Hiring extra BH workforce.
- Addition of multidisciplinary team meetings.
- Addition of a care registry and a care manager.
- Addition of brief psychotherapy interventions.
- Implementation of active depression screening of the primary care clinic’s patient population.
- Implementation of active screening of patients who are susceptible to mental illness.

1. **If yes to #3, please discuss some of the advantages and barriers to the change(s) you are considering.**

Examples of advantages of the changes suggested in question 3:

- Improving efficiency with existing resources.
- Improving quality of care.
- Improving patient safety.
- Promoting population health.
- Promoting proactive approach to care.
- Enhancing the biopsychosocial approach to care.

Examples of barriers to the changes suggested in question 3:

- The absence of a budget or incentives, such as challenges of funding a full-time care manager or the cost of embedding and utilizing a care registry.
- Lack of mechanisms for reimbursement for some proposed BH services.
- Reimbursement being dependent on individual provider billing.

**Teaching Instructions for Components of Integrated Care:**

E-Consults:

Please ensure that the learners are familiar with the definition of E-consults. As described in Appendix A, an E-consult is “an asynchronous communication between healthcare providers that occurs within a shared electronic health record (EHR) or secure Web-based platform.”

Below are two E-consult cases that you may use in case you do not receive any E-consults during a learner’s rotation. You may email the learner one of the cases and ask them to respond as if they are replying to a primary care provider (PCP)’s message on the patient’s electronic medical record.

Case #1-

- PCP: A 58-year-old woman with depression, anxiety, insomnia, no medical conditions, and normal TSH. An outside provider tried quetiapine, which made her groggy. She experienced dizziness on gabapentin 100mg. I started her on fluoxetine 20mg less than two weeks ago. Today she came back for follow up and reported no improvement of her symptoms. Her appetite is poor and she has lost weight. What would you recommend?
- Consultant: Thanks for consulting me on this case. In my opinion there are two different ways to approach this case:

Approach #1: Because it has been less than two weeks since the patient was started on fluoxetine, it would make sense to wait for at least two more weeks before considering switching to a different antidepressant, unless she has side effects from the fluoxetine. Some of the common side effects of fluoxetine include nausea, diarrhea, increased anxiety, and insomnia. It is notable that most of these side effects resolve with continued medication use.

Approach #2: Considering the patient’s poor appetite, weight loss, and insomnia, which are most likely secondary to depression and anxiety (assuming potential medical etiologies such as malignancies, autoimmune disorders, and obstructive sleep apnea have been ruled out), it would be appropriate to switch from fluoxetine to mirtazapine. Mirtazapine would help with depression and anxiety as well as poor appetite, weight loss, and insomnia. You may start at 15mg at bedtime and increase to 30mg in 1-2 weeks based on response and tolerability. Monitor for common side effects, including xerostomia and constipation. Other side effects such as drowsiness and increased appetite would likely benefit this patient.

I hope this is helpful. Please let me know if you have any questions.

Case #2-

- PCP: A 45-year-old man with depression and generalized anxiety disorder on vilazodone that was started by an outside provider. Insurance no longer covers vilazodone. What alternative medication would you recommend? Medical history is positive for HTN, chronic pain, vasculitis, Wegener’s disease, vitamin D deficiency.
- Consultant: Thanks for consulting me on this case. Vilazodone is a serotonin partial agonist and reuptake inhibitor with minimal or no effect on reuptake of norepinephrine or dopamine. Therefore, its mechanism of action differs to some extent from SSRIs and SNRIs.

Considering the patient’s chronic pain, it would be appropriate to try an SNRI (venlafaxine or duloxetine) if he has not tried either of these medications and if his blood pressure is well controlled (SNRIs can increase blood pressure, so monitoring is necessary.)

Regarding vitamin D deficiency, the patient would benefit from education on the role of vitamin D deficiency in depression and the importance of treatment adherence to ensure adequate treatment.

Regarding autoimmune diseases (vasculitis, Wegener’s disease), chronic inflammation may lead to depression. Therefore, effective treatment of the autoimmune diseases is important in managing depression. Some research studies have shown that N-Acetyl Cysteine helps treat depression in patients with autoimmune disease. If the patient is interested in trying this medication, we can provide instructions on how to take it.

If the patient has already tried and failed SNRIs, details of past medication trials are necessary before making other recommendations. In that case, the patient might benefit from further assessment by one of our psychiatrists.

Curbside Consults:

Please ensure that the learners are familiar with the definition of curbside consults. As described in Appendix A, a curbside consult is “an informal process whereby a physician obtains information or advice from another physician to assist in the management of a particular patient. The consultant is generally unfamiliar with the patient and has not reviewed the patient's chart or examined the patient.”

Warm Handoff:

Please ensure that the learners are familiar with the definition of warm handoffs. As described in Appendix A, a warm handoff is “a handoff that is conducted in person, between two members of the health care team, in front of the patient (and family if present).”
