# Supplementary material for: Integrated Behavioral Health: A Curriculum for Residents in Rural and Community Psychiatry
Source: MedEdPORTAL. 2024 Dec 20;20:11468. doi: 10.15766/mep_2374-8265.11468 (PMC11659397; doi:10.15766/mep_2374-8265.11468)
Supplement: Supplementary file 1 — Background for Facilitators.docxLearner Guide.docxSession 1 Facilitator Guide.docxSession 2 Facilitator Guide.docxSession 3 Facilitator Guide.docxSession 4 Facilitator Guide.docxFacilitator Guide Slides.pptxSimulation Scenario.docxEvaluation Survey.docx [file mep_2374-8265.11468-s001.zip › I. Evaluation Survey.docx]

**Appendix H**

**Evaluation Survey**

**Rate your perceived level of proficiency in each of the following objectives before and after your rotation:**

| **Before the rotation** | Not at all Proficient-1 | Somewhat Proficient-2 | Moderately Proficient- 3 | Very Proficient-4 | Extremely Proficient-5 |
| --- | --- | --- | --- | --- | --- |
| I was able to compare different models of BH* integration (Coordinated care, Co-located care, Integrated/reveres  integrated care, Collaborative Care Model, BH consultation). |  |  |  |  |  |
| I was able to critically appraise clinical practice, using my knowledge of different models of behavioral health integration. |  |  |  |  |  |

| **After the rotation** | Not at all Proficient-1 | Somewhat Proficient-2 | Moderately Proficient- 3 | Very Proficient-4 | Extremely Proficient-5 |
| --- | --- | --- | --- | --- | --- |
| I am able to compare different models of BH integration (Coordinated care, Co-located care, Integrated/reveres  integrated care, Collaborative Care Model, BH consultation) |  |  |  |  |  |
| I am able to critically appraise clinical practice, using my knowledge of different models of behavioral health integration. |  |  |  |  |  |

*BH: behavioral health
