# Supplementary material for: Integrated Behavioral Health: A Curriculum for Residents in Rural and Community Psychiatry
Source: MedEdPORTAL. 2024 Dec 20;20:11468. doi: 10.15766/mep_2374-8265.11468 (PMC11659397; doi:10.15766/mep_2374-8265.11468)
Supplement: Supplementary file 1 — Background for Facilitators.docxLearner Guide.docxSession 1 Facilitator Guide.docxSession 2 Facilitator Guide.docxSession 3 Facilitator Guide.docxSession 4 Facilitator Guide.docxFacilitator Guide Slides.pptxSimulation Scenario.docxEvaluation Survey.docx [file mep_2374-8265.11468-s001.zip › _Educational Summary Report_11468.pdf]

MedEdPORTAL® The AAMC Journal of Teaching and Learning Resources

Original Publication Open Access

Integrated Behavioral Health: A Curriculum for Residents in Rural and Community Psychiatry

Poonè Haghani Tehrani, MD*, Kelsey J. Sala-Hamrick, PhD, Sushilla Knottenbelt, PhD, John P. Sánchez, MD, Julie G. Salvador, PhD

*Corresponding author: phaghanitehrani@salud.unm.edu

Abstract

Introduction: Mental health and substance use disorders are common in the United States; however, only a portion of adults with these conditions receive treatment. Recent recommendations include using integrated behavioral health (IBH) models to increase patient access to care. Despite IBH's effectiveness, few psychiatry residents are trained in it. Considering the scarcity of evaluated curricula on IBH, we created a curriculum to teach different IBH models to psychiatry residents. **Methods:** The curriculum was developed using the constructivism theoretical framework and aligned with the principles of competency-based medical education. The learning activities allowed learners to apply knowledge relevant to IBH models to critically appraise a clinical scenario while practicing different components of IBH, such as electronic consults. More specifically, the curriculum assignment prompted residents to examine a clinical practice, identify the model, make recommendations for changes, and discuss the advantages and barriers of the proposed changes. We employed Kirkpatrick model levels 1 and 2b to evaluate the curriculum. **Results:** Thirty-three residents participated in this curriculum. Eleven residents completed the assignment, which was qualitatively coded to evaluate their learning. Results indicated that the participants were able to compare different IBH models and critically appraise clinical practice using knowledge of those models. Twenty-two additional residents completed an anonymous retrospective pre- and postrotation survey on their perceived level of proficiency. Survey results showed improved perceived level of proficiency at rotation completion. **Discussion:** The developed curriculum was successful in teaching residents to acquire and apply knowledge relevant to IBH.

Keywords
Integrated Psychiatry, Primary Care Integration, Teaching Curriculum, Resident Training, Rural Behavioral Health, Underserved Populations, Case-Based Learning, Community-Based Health Care, Rural Health Care & Training, Integrated Behavioral Health

Educational Objectives

By the end of this activity, learners will be able to:

1. Compare different models of behavioral health integration.
2. Critically appraise clinical practice, using their knowledge of different models of behavioral health integration.

Introduction

Mental health and substance use disorders are prevalent in the United States. According to the 2021 National Survey on Drug Use and Mental Health, one in three US adults experience a substance use disorder or mental illness each year.[1] Despite recent national attention, significant unmet needs for mental health care continue to exist. In 2016, only 1.5% of adults with a substance use disorder received any treatment, and less than 1% received treatment in a specialty facility.[2] Among adults with mental illness, only 14.4% received any type of mental health treatment.[2] Numerous barriers contribute to this gap in services, including lack of available providers, treatment cost, not knowing where to find treatment, lack of perceived need, and stigma.[2,3] Given this remarkable void in fulfilling needs, numerous policy and practice organizations have recommended primary care clinics as key targets for identifying and providing care for these patients.[4-7] Primary care clinics see 83.4% of US adults each year and can serve as a key entry point to services, where providers can identify needs, provide care, and refer to further behavioral health (BH) treatment and support services.[3]

Strong evidence exists for the use of integrated behavioral health (IBH) models in primary care clinics in order to address current gaps in mental health and substance use treatment

Citation:
Haghani Tehrani P, Sala-Hamrick KJ, Knottenbelt S, Sánchez JP, Salvador JG. Integrated behavioral health: a curriculum for residents in rural and community psychiatry. *MedEdPORTAL*. 2024;20:11468.
https://doi.org/10.15766/mep_2374-8265.11468

Copyright © 2024 Tehrani et al. This is an open-access publication distributed under the terms of the Creative Commons Attribution license.

services, improve patient physical and mental health outcomes, and reduce health care costs. Integrated care refers to "any attempt to fully or partially blend behavioral health services with general and/or specialty medical services."[8] Key components of IBH models include a multidisciplinary care team (including psychiatrists, psychologists, case managers, nurses, primary care providers [PCPs], and patients and families), a clinical setting where collaboration among team members is possible, and implemented clinical processes (screenings, risk protocols, identification of level of care needed, treatment, monitoring, and referral processes).[9]

A large body of research demonstrates the effectiveness of BH care integration. IBH models have been shown to increase access to BH care, improve quality of care, improve patient and provider satisfaction, reduce stigma, and decrease cost of care.[10,11] These models can leverage an established and trusting relationship with a PCP to promote the early identification of mental health and substance use needs and reduce patients' fear of stigma associated with seeking BH care. To this end, many studies have shown that integrated care is associated with lower no-show rates.[10-12] Moreover, utilizing IBH models promotes the use of biopsychosocial and patient-centered approaches to care, which are particularly important when treating patients from historically underserved and socioeconomically disadvantaged backgrounds. For example, utilizing multidisciplinary treatment teams allows for holistic assessment and treatment of patients, including the cultural, social, psychological, and biological aspects of their lives that may be impacting their current health, functioning, and ability to adhere to treatment.[10,12]

Psychiatrists can play a large role in implementing IBH. According to the Institute of Medicine, psychiatrists and psychiatric organizations are tasked with improving the quality of mental health and substance use care in their daily practice.[13] In alignment with these recommendations, key roles of psychiatrists on IBH teams include ensuring care is patient centered, assessing for biopsychosocial and cultural factors that may impact patients' conditions and treatment, implementing the use of valid and reliable questionnaires to assess and track care outcomes, and ensuring effective collaboration with PCPs (e.g., consulting with PCPs on diagnoses, how mental health and substance use conditions may impact other comorbidities, and recommending treatment plans).[13] In addition to IBH's positive impact on patient care, both primary and BH providers report a high level of satisfaction with and value for IBH services.[10-12] One study showed that IBH provides a context for warm handoffs where BH providers triage patients and make recommendations, while PCPs reported that it frees up their time.[12]

Despite the effectiveness of integrated primary care models, their implementation has been slow. For example, rates of PCPs utilizing evidence-based IBH models are low, less than half of PCPs colocate with a BH provider, and mental health screening rates in primary care clinics are less than 5%.[3,14-16] One potential reason for this lack of uptake is the relatively limited training that psychiatry residents and other clinicians receive on IBH models and their advantages. While some training programs exist in pediatric primary care settings,[17,18] to date there are no peer-reviewed and published curricula to support residents in learning and implementing IBH models.

Currently, there is a small number of studies on integrated care education for various groups of learners, some of which have shown positive evaluation from learners. These include a brief collaborative care curriculum for first- to fourth-year psychiatry residents,[19] a brief rotation for third-year psychiatry and family medicine residents in the family medicine clinic at five different institutions (no data on the outcome of this curriculum were provided),[20] a competency-based curriculum on IBH training for first- to third-year family medicine residents across multiple programs,[21] and a comprehensive program designed for master's-level social work students.[22] These studies offer promising data on curricula related to IBH; however, they vary with respect to the targeted group of learners, curriculum design, and evaluation. Additionally, some of them have not assessed the outcomes of their curricula.[23] Therefore, it is not possible to draw definitive conclusion regarding IBH curriculum design. Notably, the existing publications primarily concentrate on developing clinical skills as consultants in integrated settings, without placing significant emphasis on familiarity with various IBH models. Moreover, most of these studies have not published their curricula, including details of implementation.

To address these gaps, we created a curriculum in the context of newly developed IBH services at a university-affiliated hospital to teach different models of IBH to psychiatry residents. The goal was for residents to acquire and apply knowledge relevant to IBH.

Methods

Curriculum Development

The curriculum was developed using the constructivism theoretical framework. This view of learning assumes that learners, as active participants in their learning, constantly strive to make meaning of their learning experiences. This

Copyright © 2024 Tehrani et al. This is an open-access publication distributed under the terms of the Creative Commons Attribution license.

process is affected by the individual learner's existing knowledge foundations and cognitive operations, as well as the learning activities in which they engage.[24] This approach aligns with the Accreditation Council for Graduate Medical Education (ACGME) competency-based and learner-centered guidelines.[25]

The curriculum-targeted learners were second-year psychiatry residents, who participated in the learning activities 1 day a week as part of a preexisting 1-month rural and community psychiatry rotation. The learners had no prior formal training in IBH in their residency training.

To familiarize themselves with foundational knowledge about IBH, facilitators used the background for facilitators (Appendix A) and the learner guide (Appendix B).

The curriculum's learning activities were organized in four main categories: (1) acquiring information and ideas, (2) experience, (3) reflection, and (4) assessment of the learners and the curriculum (Figure). A literature review of different IBH models and a clinical scenario provided the educational context for acquiring and applying knowledge relevant to IBH. While not required, we offered an optional 4-day rotation as an opportunity to observe and experience practice of IBH within a family practice clinic.[26]

As outlined in the Figure, for acquiring information and ideas, the residents reviewed the learner guide (Appendix B). This document included the curriculum's learning goals and objectives; an overview of the importance and different models of IBH, including major elements of the collaborative care model; and the curriculum assignment and citations for the content on IBH. As described in the session 1 facilitator guide (Appendix C), the facilitator provided this document to the participating resident right before or on the first day of the rotation. The second phase of acquiring information and ideas took place mid-rotation, when the resident reviewed the literature on different IBH models, including their advantages and limitations. This is further explained in conjunction with the learning activities described in the following paragraphs.

For experience, we defined several learning activities to be completed in sessions 1-4, along with a reflection exercise in session 4. Session 1 (Appendix C) began with a discussion of the learning goal and objectives, providing an opportunity for residents to participate in planning their learning. This was followed by an overview of the learning activities. Additionally, the communication of the rotation's expectations, ground rules for mutual feedback (e.g., timing, frequency, and aims for feedback), introduction to the clinic staff and providers, and orientation to the clinic physical space took place at the beginning of this session.

In addition to partaking in setting goals, the resident observed the clinical practice and participated in components of integrated care during session 1. These components included electronic consults (E-consults), curbside consults, and warm handoffs, which the facilitator discussed with the resident as described in Appendix C. Appendix C also provided examples for simulated E-consults to be used if the integrated team did not receive an E-consult during the resident's time at the rotation site.

In session 2, the main learning activity was to answer two questions referred to as assignment questions 1 and 2 in the curriculum outline and the supporting material. These questions prompted the resident to discuss the advantages and limitations of the observed integrated practice and to identify the model(s) of this practice. The session 2 facilitator guide (Appendix D) included instructions for leading this session.

In session 3, the primary focus was to review the resident's responses to assignment questions 1 and 2; discuss the details, including different models of IBH; and address possible misconceptions. The session 3 facilitator guide (Appendix E) provided details for conducting this discussion.

Session 4 contained three key activities: answering assignment questions 3 and 4, reviewing and discussing the resident's response to these questions, and the reflection exercise. Assignment questions 3 and 4 prompted the resident to examine whether they would consider making changes to the practice they had observed and, if so, to discuss the advantages and barriers of the changes they were considering. The detailed guide for conducting this discussion was included in the session 4 facilitator guide (Appendix F).

Appendix G provided supplementary PowerPoint slides for Appendices C-F to enhance teaching.

Curriculum Implementation

The curriculum was implemented at a family practice clinic (University of New Mexico Sandoval Regional Medical Center) where psychiatric services were integrated to improve the quality of and access to care for the underprivileged patient population of the clinic within the limits of the existing resources. The curriculum was executed in 2017 with ongoing iterations (except in 2020, due to the COVID-19 pandemic) guided by the on-site psychiatrist. It could be implemented in any IBH setting, regardless of the model of integration. When implementing

Copyright © 2024 Tehrani et al. This is an open-access publication distributed under the terms of the Creative Commons Attribution license.

Acquiring Information and Ideas:

- At the beginning of the rotation: reading the overview of the importance and different models of integrated behavioral health, including major elements of the collaborative care model.
- Mid-rotation: reviewing the literature on different integrated behavioral health models, including their advantages and limitations.

Experience:

Learning activities:

- Discussion of learning goals, objectives, and activities.
- Observation of the clinical practice.
- Participation in and discussion of the components of integrated care.
- Completion of the assignment questions.
- Discussion of the assignment questions.

Reflection:

- How did this experience influence your view of outpatient behavioral health practices and/or your interests?
- What are you interested in learning next?

Assessment of the Learners and the Curriculum:

Mid-rotation:

1. Please discuss some of the advantages and limitations of the current practice of integrated behavioral health at [*the name of the rotation site*].
2. Which model(s) of care is (are) being practiced at [*the name of the rotation site*]? Provide evidence to support your answer.

At the end of the rotation:

3. Imagine you are the psychiatrist [*or the behavioral health provider*] conducting the behavioral health services at [*the name of the rotation site*]. Would you consider making changes to the current practice?
4. If yes to 3, please discuss some of the advantages and barriers of the change(s) you are considering.

Figure. Curriculum outline.

this curriculum in an IBH setting, it was crucial not to disclose the specific IBH model. This approach allowed learners the opportunity to identify the model as part the curriculum assignment. Additionally, Appendix H offered a supplementary simulation scenario for implementation of this curriculum outside of a clinical setting. Furthermore, the curriculum could be completed in two instead of four sessions. The instructions for this were included in Appendix C.

Curriculum Evaluation

To evaluate the curriculum's effectiveness in accomplishing the Educational Objectives (EOs), we employed levels 1 and 2b of the Kirkpatrick model, a four-level model originally created for industry training evaluation but widely used in educational assessments. These include medical education, which has adopted a modified version of the model. Consistent with Kirkpatrick level 1, we gauged learners' perception of the curriculum's effectiveness. Based on Kirkpatrick level 2b, we assessed the curriculum's impact on learners' knowledge and skills.[27] Data from the first seven classes of residents who participated in the curriculum were used to evaluate the curriculum. An average of 9.6 residents per year participated in this rotation. Only residents who participated in at least two sessions of the rotation and completed the assignment were included (total of 33 residents).

For the first three classes of residents who participated in the rotation ($n = 11$), residents' responses to the curriculum assignment were used to assess the effectiveness of the curriculum. This was consistent with the principle of assessment for learning instead of assessment of learning, which fosters a growth mindset.[28] An inductive approach to content analysis was used to code data focused on the EOs. NVIVO qualitative software was used to code resident responses and to help

Copyright © 2024 Tehrani et al. This is an open-access publication distributed under the terms of the Creative Commons Attribution license.

identify overarching themes that emerged directly from resident responses to the curriculum assignment.[29,30] Using the EOs and resident assignment as a coding guide, the senior author, Julie G. Salvador, a trained qualitative researcher, conducted initial coding of the written responses and developed preliminary theme labels. Julie G. Salvador and Poonè Haghani Tehrani (lead author) then jointly reviewed the coded statements and theme labels to come to a final agreement on placement of codes and meaningful theme labels. Themes were organized under each area according to the focus of learner responses relating to advantages, limitations, and suggestions for improvement identified by learners at the provider, patient, or general health care system level.

For the last four classes of residents who participated in the rotation ($n = 22$), an anonymous retrospective pre- and postrotation survey (Appendix I) was implemented. The survey assessed the residents' perceived level of proficiency in each of the curriculum EOs before and after the rotation. University of New Mexico Institutional Review Board (IRB) exemptions were obtained.

Results

Thirty-three residents participated in this rotation. To help evaluate the curriculum, 11 residents completed the qualitative curriculum assignment, and the remaining 22 completed a quantitative survey. Results from both the qualitative and quantitative evaluation processes are presented below.

Qualitative Evaluation

The resident responses to the assignment were qualitatively analyzed to evaluate the accomplishment of the curriculum EOs: (1) how well residents were able to compare different models of BH integration and (2) how well residents were able to critically appraise clinical practice, using their knowledge of different models on BH integration. Regarding EO 1, the results of this qualitative analysis showed that residents were able to compare different models of IBH. As part of the assignment, residents were prompted to review the literature on different models of IBH and discuss what model or models were being practiced at the rotation site. In response, residents identified general features of IBH models. Collaborative care was referred to most often, with learners describing this model as led by a PCP and supported by care managers as liaisons between the patient, the PCP, and the BH professional. Fundamental aspects of this model identified included that it is evidence based and team driven, uses multidisciplinary teams and problem-based referral, is population based, and uses measurement-guided treatment.

A second model identified was colocation. Learners described this model as patients seeing both the PCP and a BH expert, with frequent communication between providers on shared goals of the patient. Regarding EO 2, residents demonstrated the ability to critically appraise clinical practice using their knowledge of different models of BH integration. In their responses to the curriculum assignment, residents discussed numerous advantages, limitations, and recommendations for improvement of the integrated model at the rotation site. These responses are narratively described below and reflected in Tables 1-3. For each area (advantages, limitations, and recommendations for improvement), responses focused on three general categories: impact on clinicians and providers, impact on patients, and impact on health care system and processes.

Table 1 shows residents' responses regarding the advantages of integrated practice. At the clinician/provider level, these advantages included improved communication and collaboration resulting from integrated care, such as the ability to have curbside consultations and better support between the PCP and psychiatrist. At the patient level, themes included enhanced access to and quality of care, and improved patient outcomes. Residents also reported that integrated care helped reduce stigma for patients about receiving psychiatric treatment. For the health care system, residents reported that integrated care helped reduce costs and liability for providers and that the model could be implemented in a wide range of venues.

Table 2 presents residents' responses regarding the limitations of integrated practice. At the clinical/provider level, these limitations included challenges with time management and spending time with lower-acuity patients who could be effectively managed by midlevel BH providers. At the patient level, residents reported that IBH programs worked best in contexts where the patient had multiple comorbidities and psychiatric issues were not the primary diagnosis. At the health care system level, residents noted the need to develop protocols to support implementation.

Table 3 presents residents' recommendations for improvement of integrated practice. At both the clinical/provider level and the patient level, more education was recommended, aimed at helping PCPs to identify and refer patients appropriately and to enhance awareness and comfort for patients to access BH services. At the health care system level, recommendations included adding other BH services (e.g., group therapy) and financial incentives to encourage the implementation of IBH.

Copyright © 2024 Tehrani et al. This is an open-access publication distributed under the terms of the Creative Commons Attribution license.

Table 1. Residents' Responses on Advantages of the Integrated Practice at the Rotation Site (*N* = 11)

	Advantages	
**Category**	**Theme**	**Description**
Clinicians/providers	Improved communication and collaboration	The learners pointed out the advantages of shared electronic medical records and face-to-face discussions of an appropriate treatment plan for patients, including challenging patients, as well as avoiding medication interactions.
Patients	Improved access to care	Through an easier referral process, decreased likelihood of referral failure, increased capacity for the psychiatrist to see patients, and reduced wait time.
	Improved quality of care	Statements describing better coordination of care, better continuity of care, and an easier transition back to the primary care provider once acuity is resolved. Patients were also more likely to have completed a mental health and medical assessment, providers were more accountable to patients and each other, care was measurement based, and providers each benefited from education from one another.
	Improved patient treatment adherence and retention	Statements indicating that because the model improves quality of behavioral health care, it supports greater reinforcement for treatment adherence and improves retention in care.
	Improved clinical outcomes	Statements indicating that the model improves patient outcomes through improved access to quality care, including medication awareness between providers (as noted above).
	Stigma reduction	Statements that the shared physical space helps patients feel less nervous about accessing this care and can destigmatize obtaining psychiatric services and that patients are more familiar with providers in integrated practice settings.
Health care system and processes	Costs/liability	Statements indicating reduced administrative costs resulting in lower overall cost for the patient, payers (e.g., insurance), and facilities, as well as reduced liability for both primary care providers and behavioral health providers.
	Improved implementation	Responses reflected that integrated behavioral health in general could be implemented in a range of settings, including those with limited resources, and that the model supported more effective implementation of quality improvement interventions to promote overall wellness.

Quantitative Evaluation

Twenty-two residents completed a retrospective pre- and postrotation anonymous survey (survey response rate: 100%) where they rated their proficiency in comparing different models of IBH (EO 1) and in critically appraising clinical practice using their knowledge of different models of IBH (EO 2) on a 5-point scale (1 = *not at all proficient*, 5 = *extremely proficient*). The mean rating of resident proficiency in comparing different models of IBH increased from 1.9 to 4.1. The mean rating of their ability to critically appraise clinical practice using their knowledge of different models of IBH improved from 1.6 to 3.9.

Discussion

Considering the evidence for the effectiveness of IBH in fulfilling health care goals of improving quality of care and health of populations while reducing the cost of care,[8-12,31] it seems reasonable to anticipate an increasing demand for psychiatrists, other BH professionals, family medicine physicians, and pediatricians to be part of developing or operating in IBH settings. Therefore, it is crucial that these groups of health care professionals are familiar with IBH models. This is reflected in the inclusion of "understanding of models of integrated multidisciplinary mental health and primary care" and serving "as a leader of integrated teams of implementation projects" in the ACGME milestones for psychiatry residents.[25]

To address the gap in learners' familiarity with different IBH models, we developed and evaluated the curriculum detailed herein. The results revealed the effectiveness of our brief and innovative educational resource. Given the constraints of the overarching rotation, including considerations of timing and duration, the curriculum's learning activities were designed to

Table 2. Residents' Responses on Limitations of the Integrated Practice at the Rotation Site (*N* = 11)

	Limitations	
**Category**	**Theme**	**Description**
Clinicians/providers	Challenges with time management	Due to not knowing how many new patients a provider might see in a day (i.e., too many or too few).
	Seeing patients with lower acuity	The psychiatrist would not have control over referrals and might see many patients with lower mental health needs.
	The need for midlevel providers	For triaging patients appropriately and ensuring psychiatrists could work at the top of their licensure.
Patients	Works in selective populations	Patients with multiple medical comorbidities and where psychiatric issues were not the patient's primary problem.
Health care system and processes	The need to develop new processes and protocols	To support implementation of integrated behavioral health. For example, provider's billing and the need to establish treatment team meetings between providers and staff to discuss patient care.

Copyright © 2024 Tehrani et al. This is an open-access publication distributed under the terms of the Creative Commons Attribution license.

Table 3. Residents' Responses on Recommendations for Improvement of the Integrated Practice at the Rotation Site ($N = 11$)

Category	Recommendations for Improvement	
	Theme	Description
Clinicians/providers	Improve education	Education to help primary care providers identify and treat patients directly who have low-level mental health needs and to improve provider skills to identify and refer patients with higher acuity.
Patients	Improve education	More information about behavioral health services to build awareness and openness to how to utilize these services.
Health care system and processes	A screening process to identify patients with behavioral health needs	Developing a screening process and potentially a care registry for this information.
	More midlevel providers	Needed for triaging and providing care for persons at lower levels of acuity (also coded as a limitation in Table 2).
	Access to group therapy	For patients who did not need individualized therapy, ensuring their needs were met while using psychiatric time wisely.
	Financial incentives	To encourage providers to implement this model in practice.

cater to early-stage learners while serving as an assessment for learning. Significantly, this design was shown to be effective in achieving the curriculum EOs, as the learners were able to acquire adequate knowledge of different models of IBH, apply that knowledge to a comparison of various existing models, and critically appraise the rotation site model. Our curriculum design, tailored to early-stage learners, provides them with an opportunity to explore their interest in IBH. Additionally, the curriculum's brevity enhances its feasibility.[21]

We encountered barriers during the curriculum's implementation and data collection. One challenge was completing IRB approval after the enrolled residents had graduated. Consequently, we were unable to obtain consent to include quotes from their responses to the curriculum assignment. Another obstacle arose from the fact that second-year psychiatry residents' training in our program predominantly focuses on inpatient and psychiatric emergency services. As a result, some residents found it challenging to shift from fast-paced, high-acuity settings to outpatient care, and some reported discomfort commuting to the rotation site. Moreover, some classes were encouraged to take leave during their rural and community rotation, which encompassed our curriculum. Also, with the onset of the COVID-19 pandemic, it became more likely that backup on-call residents would be called in. These factors impacted the number of residents who completed this rotation and, therefore, our sample size. A unique challenge in teaching different IBH models is the use of the term *integrated* to refer to higher levels of integration, rather than encompassing the full spectrum of integrated care models. This deserves attention in future studies on IBH models and the associated educational experiments.

One essential element of this curriculum's implementation was its location at a newly developed university-affiliated hospital in an underserved community. While this provided institutional support for the development of integrated services in the family practice clinic, including support for faculty time, dedicated clinical space and staff, integration of the faculty psychiatrist and residents into the clinic's physical space and providers' workstations, and acceptance of the residents in their role, it also presented challenges. Many academic institutions partner with nonacademic facilities, offering learning opportunities but potentially causing implementation challenges due to the separation of leadership and stakeholders. For instance, due to resource and workforce limitations, it was challenging to garner support for residents' quality improvement projects related to this curriculum. Also, because of the distribution of funding under the Office for Graduate Medical Education, interested residents could not return to this rotation site for an elective rotation to expand their clinical experience in integrated care.

This project had some limitations. Firstly, responses to the curriculum assignment were obtained either in person verbally or via email, potentially impacting how residents responded. Based on their preference and learning style, learners were given the choice of an in-person verbal discussion of their responses to the assignment or an email exchange. For those who discussed their responses verbally, accuracy was ensured by repeating their responses back to them and documenting them instantly. Another limitation was the variation in the number of days residents participated in the rotation, ranging from 2 to 4 days. Despite this, all residents completed the assignment and were able to critique the practiced model at the rotation site. Additionally, we had a small sample size ($n = 33$). While having a larger group of participants would have been beneficial, the rotation was part of a 1-month rural and community psychiatry track, which was an elective during the first few years of this project. Therefore, only one resident per month could opt in if they chose to participate in the rural and community track.

Our goal is to make this curriculum available to educators as part of global efforts toward IBH training. Given the scarcity of

Copyright © 2024 Tehrani et al. This is an open-access publication distributed under the terms of the Creative Commons Attribution license.

published, peer-reviewed curricula, this publication contributes to the development, implementation, and standardization of integrated care curricula. This curriculum is adaptable for utilization by psychiatry, family medicine, and pediatrics residency programs, as well as programs in BH training, including clinical psychology, social work, BH counseling, nurse practitioner, and physician assistant training programs. It can be implemented in primary care and specialty care clinics, in academic and nonacademic institutions, spanning urban and underserved areas, regardless of the type of integrated model practiced, as well as outside of clinical settings.

Appendices

A. Background for Facilitators.docx
B. Learner Guide.docx
C. Session 1 Facilitator Guide.docx
D. Session 2 Facilitator Guide.docx
E. Session 3 Facilitator Guide.docx
F. Session 4 Facilitator Guide.docx
G. Facilitator Guide Slides.pptx
H. Simulation Scenario.docx
I. Evaluation Survey.docx

All appendices are peer reviewed as integral parts of the Original Publication.

Poonè Haghani Tehrani, MD: Associate Professor, Department of Psychiatry and Behavioral Sciences, University of New Mexico Health Sciences Center

Kelsey J. Sala-Hamrick, PhD: Clinical Psychologist, Department of Psychiatry and Behavioral Sciences, University of New Mexico Health Sciences Center

Sushilla Knottenbelt, PhD: Senior Lecturer III, Department of Chemistry and Chemical Biology, University of New Mexico

John P. Sánchez, MD: Dean, Universidad Central Del Caribe School of Medicine

Julie G. Salvador, PhD: Associate Professor, Department of Psychiatry and Behavioral Sciences, University of New Mexico Health Sciences Center

Acknowledgments
The BNGAP Academic Medicine Writing Fellowship, sponsored through School of Medicine at the University of New Mexico, provided valuable support for this work.

Disclosures
None to report.

Funding/Support
None to report.

Ethical Approval
The University of New Mexico Health Sciences Center Human Research Protection Program deemed further review of this project not necessary.

References

1. Substance Abuse and Mental Health Services Administration. *Key Substance Use and Mental Health Indicators in the United States: Results From the 2021 National Survey on Drug Use and Health*. Center for Behavioral Health Statistics and Quality, Substance Abuse and Mental Health Services Administration; 2022. HHS publication PEP22-07-01-005. Accessed December 4, 2024. https://www.samhsa.gov/data/sites/default/files/reports/rpt39443/2021NSDUHFFRRev010323.pdf
2. Park-Lee E, Lipari RN, Hedden SL, Kroutil LA, Porter JD. Receipt of services for substance use and mental health issues among adults: results from the 2106 National Survey on Drug Use and Health. Substance Abuse and Mental Health Services Administration. September 2017. Accessed December 4, 2024. https://www.samhsa.gov/data/sites/default/files/NSDUH-DR-FFR2-2016/NSDUH-DR-FFR2-2016.htm
3. Kyanko KA, Curry LA, Keene DE, Sutherland R, Naik K, Busch SH. Does primary care fill the gap in access to specialty mental health care? A mixed methods study. *J Gen Intern Med*. 2022;37(7):1641-1647. https://doi.org/10.1007/s11606-021-07260-z
4. Promoting access to behavioral health care. Association of American Medical Colleges. Accessed December 4, 2024. https://www.aamc.org/about-us/mission-areas/health-care/behavioral-health-care
5. National Academies of Sciences, Engineering, and Medicine. *Implementing High-Quality Primary Care: Rebuilding the Foundation of Health Care*. National Academies Press; 2021. Accessed December 4, 2024. https://doi.org/10.17226/25983
6. American Academy of Pediatrics recommends integrating mental and behavioral health surveillance and supports into all office visits. American Academy of Pediatrics. August 2, 2021. Accessed December 4, 2024. https://www.aap.org/en/news-room/news-releases/aap/2021/american-academy-of-pediatrics-recommends-integrating-mental-and-behavioral-health-surveillance-and-supports–into-all-office-visits/
7. Behavioral health integration in physician practices. American Medical Association. Updated June 14, 2024. Accessed December 4, 2024. https://www.ama-assn.org/delivering-care/public-health/behavioral-health-integration-physician-practices
8. Collaborative/integrated care: improving access to mental health services and the overall health of patients. American Psychiatric

Copyright © 2024 Tehrani et al. This is an open-access publication distributed under the terms of the Creative Commons Attribution license.

Association. Accessed December 4, 2024. https://www.psychiatry.org/psychiatrists/practice/professional-interests/integrated-care

9. Kwan BM, Valeras AB, Brown Levey S, Nease DE, Talen ME. An evidence roadmap for implementation of integrated behavioral health under the Affordable Care Act. *AIMS Public Health*. 2015; 2(4):691-717. https://doi.org/10.3934/publichealth.2015.4.691
10. Sunderji N, Ion A, Ghavam-Rassoul A, Abate A. Evaluating the implementation of integrated mental health care: a systematic review to guide the development of quality measures. *Psychiatr Serv*. 2017;68(9):891-898. https://doi.org/10.1176/appi.ps.201600464
11. Dale H, Lee A. Behavioural health consultants in integrated primary care teams: a model for future care. *BMC Fam Pract*. 2016;17:97. https://doi.org/10.1186/s12875-016-0485-0
12. Muse AR, Lamson AL, Didericksen KW, Hodgson JL. A systematic review of evaluation research in integrated behavioral health care: operational and financial characteristics. *Fam Syst Health*. 2017;35(2):136-154. https://doi.org/10.1037/fsh0000276
13. Pincus HA, Page AEK, Druss B, Appelbaum PS, Gottlieb G, England MJ. Can psychiatry cross the quality chasm? Improving the quality of health care for mental and substance use conditions. *Am J Psychiatry*. 2007;164(5):712-719. https://doi.org/10.1176/appi.ajp.164.5.712
14. Akincigil A, Matthews EB. National rates and patterns of depression screening in primary care: results from 2012 and 2013. *Psychiatr Serv*. 2017;68(7):660-666. https://doi.org/10.1176/appi.ps.201600096
15. Bishop TF, Ramsay PP, Casalino LP, Bao Y, Pincus HA, Shortell SM. Care management processes used less often for depression than for other chronic conditions in US primary care practices. *Health Aff (Millwood)*. 2016;35(3):394-400. https://doi.org/10.1377/hlthaff.2015.1068
16. Richman EL, Lombardi BM, Zerden LD. Mapping colocation: using national provider identified data to assess primary care and behavioral health colocation. *Fam Syst Health*. 2020;38(1):16-23. https://doi.org/10.1037/fsh0000465
17. Meyers N, Maletz B, Berger-Jenkins E, et al. Mental health in the medical home: a longitudinal curriculum for pediatric residents on behavioral and mental health care. *MedEdPORTAL*. 2022;18:11270. https://doi.org/10.15766/mep_2374-8265.11270
18. Leadership Education in Neurodevelopmental and Related Disabilities (LEND) Programs. Association of University Centers on Disabilities (AUCD). Accessed December 4, 2024. https://www.aucd.org/about-lends
19. Huang H, Barkil-Oteo A. Teaching collaborative care in primary care settings for psychiatry residents. *Psychosomatics*. 2015;56(6):658-661. https://doi.org/10.1016/j.psym.2015.03.006
20. Cowley D, Dunaway K, Forstein M, et al. Teaching psychiatry residents to work at the interface of mental health and primary care. *Acad Psychiatry*. 2014;38(4):398-404. https://doi.org/10.1007/s40596-014-0081-3
21. Martin MP, Banks E, Myerholtz L, Zubatsky M, Suri Y, Mauksch L. Preparing residents to practice integrated behavioral health care: multi-site feasibility study of a competency-based curriculum. *Int J Psychiatry Med*. 2023;58(3):201-213. https://doi.org/10.1177/00912174221086930
22. DeBonis JA, Becker MA, Yoo J, Capobianco J, Salerno A. Advancing social work education: lessons learned from piloting an integrated healthcare curriculum. *J Soc Serv Res*. 2015;41(5): 594-621. https://doi.org/10.1080/01488376.2015.1081859
23. Burruss NC, Murray C, Li W, Sowa NA. Integrated care education for general psychiatry residents in the US: a review of the literature. *Acad Psychiatry*. 2023;47(4):390-401. https://doi.org/10.1007/s40596-023-01760-2
24. Dennick R. Twelve tips for incorporating educational theory into teaching practices. *Med Teach*. 2012;34(8):618-624. https://doi.org/10.3109/0142159X.2012.668244
25. Edgar L, McLean S, Hogan SO, Hamstra S, Holmboe ES. *The Milestones Guidebook*. Accreditation Council for Graduate Medical Education; 2020. Accessed December 4, 2024. https://www.acgme.org/globalassets/milestonesguidebook.pdf
26. Vimalananda VG, Gupte G, Seraj SM, et al. Electronic consultations (e-consults) to improve access to specialty care: a systematic review and narrative synthesis. *J Telemed Telecare*. 2015;21(6):323-330. https://doi.org/10.1177/1357633X15582108
27. Yardley S, Dornan T. Kirkpatrick's levels and education "evidence." *Med Educ*. 2012;46(1):97-106. https://doi.org/10.1111/j.1365-2923.2011.04076.x
28. Richardson D, Kinnear B, Hauer KE, et al. ICBME Collaborators. Growth mindset in competency-based medical education. *Med Teach*. 2021;43(7):751-757. https://doi.org/10.1080/0142159X.2021.1928036
29. Thomas DR. A general inductive approach for analyzing qualitative evaluation data. *Am J Eval*. 2006;27(2):237-246. https://doi.org/10.1177/1098214005283748
30. Hsieh HF, Shannon SE. Three approaches to qualitative content analysis. *Qual Health Res*. 2005;15(9):1277-1288. https://doi.org/10.1177/1049732305276687
31. Smith CD, Balatbat C, Corbridge S, et al. Implementing optimal team-based care to reduce clinician burnout. National Academy of Medicine. September 17, 2018. Accessed December 4, 2024. https://doi.org/10.31478/201809c

Received: February 13, 2024
Accepted: August 16, 2024
Published: December 20, 2024

Copyright © 2024 Tehrani et al. This is an open-access publication distributed under the terms of the Creative Commons Attribution license.
